# Conversion and upgrading of syringate by *Acinetobacter baylyi* ADP1

**DOI:** 10.1186/s12934-025-02839-1

**Published:** 2025-09-29

**Authors:** Heidi Tuomela, Johanna Koivisto, Elena Efimova, Suvi Santala

**Affiliations:** Faculty of Engineering and Natural Sciences, Tampere University, Hervanta Campus, Korkeakoulunkatu 8, 33720 Tampere, Finland

**Keywords:** Lignin, Syringate, *O*-demethylation, Vanillate *O*-demethylase, *Acinetobacter baylyi* ADP1, 2-pyrone-4,6-dicarboxylate

## Abstract

**Background:**

Lignin holds great potential as an abundant and sustainable source of aromatic compounds, offering a viable alternative to fossil-based resources for producing chemicals and materials. Biological upgrading of lignin-derived aromatics can lead to more comprehensive lignocellulose utilization, thereby enhancing the overall feasibility of production. However, exploring a broader range of potential microbial hosts, pathways, and enzymes is crucial for developing efficient conversion processes. In particular, improving the conversion of S-lignin-related aromatics, such as syringate, remains a key area for future research.

**Results:**

In this study, we aimed to investigate the conversion of S-lignin-related syringate in *Acinetobacter baylyi* ADP1 by exploiting its native vanillate demethylase, VanAB. We discovered that the wild-type strain can efficiently *O*-demethylate syringate to 3-*O*-methylgallate (3MGA) and then to gallate, revealing a previously unknown activity of VanAB of *A. baylyi* ADP1. Conversion dynamics and in vitro characterization showed that VanAB prefers syringate as a substrate over 3MGA. Overexpression of *vanAB* resulted in simultaneous conversion of syringate and 3MGA, but negatively impacted growth, potentially due to toxic side product formaldehyde and redox imbalance caused by high NADH consumption of the *O*-demethylation reactions. Native *vanAB* expression resulted in 3MGA accumulation if syringate was available. We took advantage of this by constructing a strain with heterologous expression of *galA*, a gallate dioxygenase from *Pseudomonas putida* KT2440, and demonstrated the conversion of the intermediate 3MGA into 2-pyrone-4,6,-dicarboxylate (PDC), a precursor for high-quality polyesters.

**Conclusions:**

In this study, we discovered a previously unknown activity of syringate conversion in *A. baylyi* ADP1. By adjusting the expression level of *vanAB*, syringate can be directed either into gallate or 3MGA, which could be further converted into PDC through the heterologous expression of *galA.* Our results further highlight the potential and versatility of *A. baylyi* ADP1 for the conversion and upgrading of lignin-related aromatic compounds.

**Supplementary Information:**

The online version contains supplementary material available at 10.1186/s12934-025-02839-1.

## Background

Lignin, comprising up to a third of lignocellulose, is a natural polymer found in all wood-like plants. It is well known for its tenacious structure that is formed by complex networks of three main subunits –*p*-hydroxyphenyl (H), guaiacyl (G) and syringyl (S) units. While lignin provides protection and support for plants, it is considered a nuisance in industries that use lignocellulose as raw material. In these industries, burning lignin for power generation is the main means to derive value from it. Therefore, upgrading lignin to valuable products could improve the economic feasibility of biobased industries and contribute to more carbon-wise use of resources. Lignin also provides a renewable source of aromatics, an alternative to current fossil-based feedstocks for chemical production.

Depolymerized lignin consists of a heterogeneous mixture of aromatic compounds, which varies across different plant types. Biological upgrading of lignin is a promising approach, as it allows these diverse aromatic compounds to be funnelled to specific, defined products. Recently, the native catabolic pathways of soil bacteria, such as *Pseudomonas putida* KT2440, *Sphingobium* sp. SYK-6, *Novosphingobium aromaticivorans* and *Acinetobacter baylyi* ADP1 have been utilized for upgrading lignin-related aromatic compounds into value-added products [1, 2]. For example, the biological production of plastic precursors such as *cis*,* cis*-muconate [3, 4], adipate [5, 6], gallate [7, 8], and 2-pyrone-4,6-dicarboxylate (PDC) [9, 10] has been extensively studied. Plastics produced from biobased precursors can have many advantages over their fossil-based counterparts, such as better biodegradability, and improved mechanical and adhesive properties [11–13]. PDC is an enticing target product for microbial valorisation of lignin [14, 15] as its chemical synthesis is very difficult, making it a so called bioprivileged compound [14]. PDC production has been demonstrated from single lignin monomers including vanillate, syringate, 4-hydroxybenzoate and *p*-coumarate, as well as from lignin derived feedstock [10, 16–22]. A significantly high titer of 99.9 g/L and productivity of 1.69 g/L/h were achieved by the expression of *vanAB* from *Pseudomonas putida* KT2440 and *ligABC* from *Sphingobium* sp. SYK-6 in *Pseudomonas putida* PpY1100 using vanillate as a substrate [9].

S-lignin constitutes a significant portion of the lignin polymer, particularly in grasses and hardwood [23]. S-lignin derived aromatics, such as syringaldehyde and syringate, are prevalent in depolymerized lignin. However, microbial strains capable of catabolizing these compounds are scarce [24, 25]. The different lignin types are distinguished by the number of methoxy groups attached to the aromatic ring. S-lignin derived aromatics are the most challenging to catabolize because they contain two methoxy groups, compared to G-lignin with one methoxy group and H-lignin, which is not methoxylated. Consequently, *O-*demethylation of these methoxy groups is a crucial step in the upper pathways of aromatic catabolism.

**Fig. 1:**
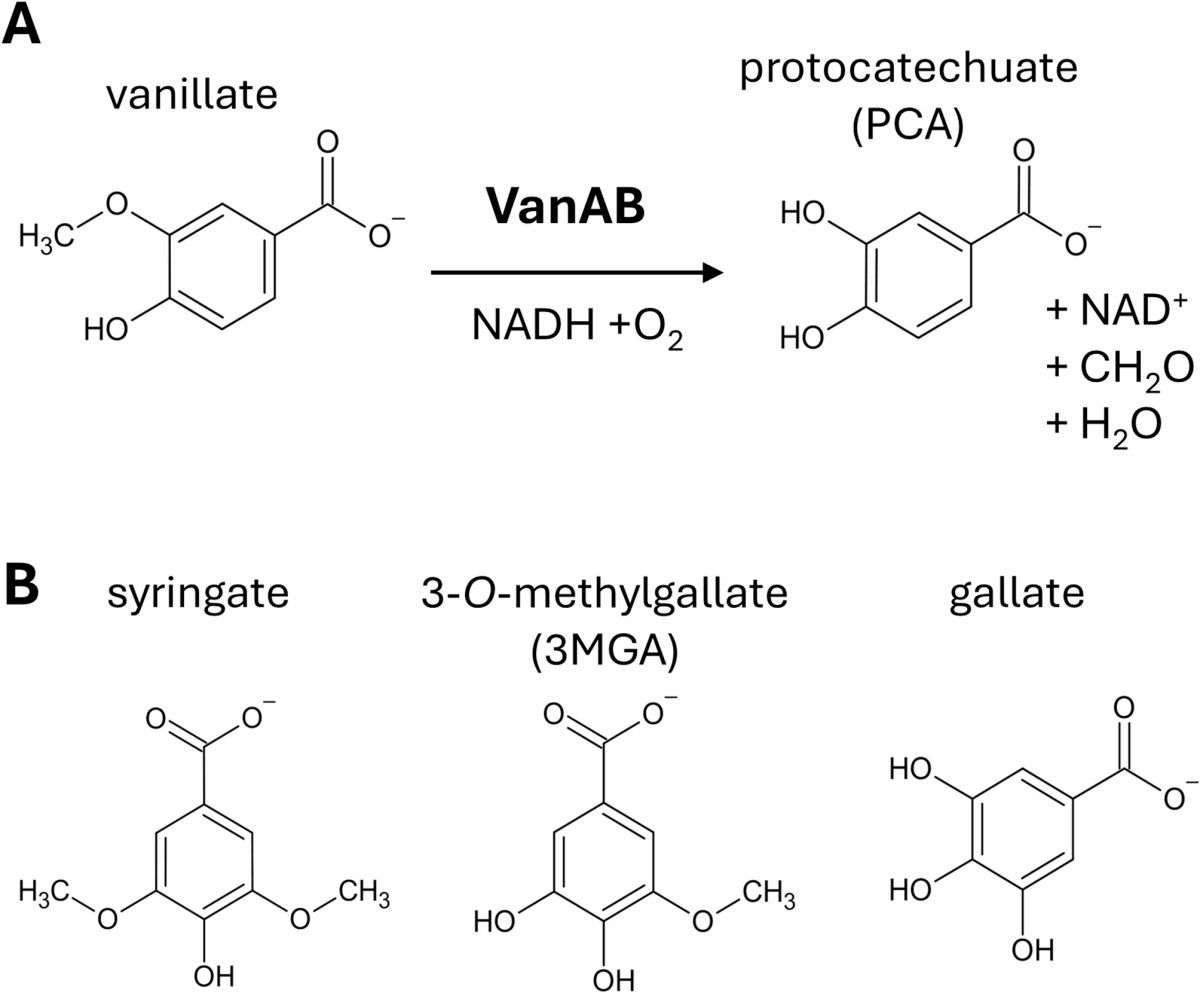
The enzymatic reaction performed by VanAB. (A) VanAB O-demethylates its substrate vanillate by using NADH and oxygen. Protocatechuate (PCA), NAD^+^, and formaldehyde are formed as products. (B) The chemical structures of syringate, 3-O-methylgallate (3MGA), and gallate. O-demethylation of syringate produces 3MGA, and further O-demethylation of 3MGA produces gallate

In aerobic microorganisms, *O-*demethylation is carried out by enzymes that can be divided into three groups: Rieske oxygenases (RO), cytochromes P450 (P450s), and tetrahydrofolate (THF)-dependent demethylases [26]. THF-dependent demethylases transfer the methyl group non-oxidatively to THF [27], whereas ROs and P450s utilize NAD(P)H as a cofactor to oxidize the methyl group into formaldehyde [26]. Formaldehyde, being highly toxic to cells, can be mitigated by more than one pathway [28]. For example, in *E. coli frmA* encodes a glutathione dependent formaldehyde dehydrogenase that is coupled to restoring NAD + into NADH [29]. The requirement for NADH increases for substrates with several methoxy groups, for which *O-*demethylating reactions have been shown to be energy-limited and cause cofactor imbalance in cells. Substrates (such as glucose) that drive primary metabolism are often required to support cell’s energy generation to obtain a balanced redox stoichiometry and sustain efficient utilization of the aromatic compounds [9, 26, 30]. Rieske oxygenases consist of a reductase, an oxygenase, and in some cases an additional ferredoxin for electron transfer. The reductase transfers electrons from NAD(P)H to the oxygenase resulting in reduction of the mononuclear iron. Binding of the substrate leads to binding of O_2_ at the iron-center, resulting in a high-valency ferryl species that can hydroxylate the substrate. The final product is obtained after rearrangement of the hydroxylated species [31]. VanAB is a Rieske oxygenase that uses vanillate as its primary substrate (Fig. 1A). Previously it has been shown that this enzyme pair can *O-*demethylate syringate into 3-*O*-methylgallate and subsequently into gallate in *Pseudomonas* sp. HR199, *Pseudomonas putida* KT2440 and *Streptomyces* sp. NL15-2 K (Fig. 1B) [30, 32–34]. Recently, in *Pseudomonas* sp. NGC7 several VanAB homologues were found, of which VanA1B1 converts specifically syringate into 3MGA [35].

Although the substrate range of VanAB in *A. baylyi* ADP1 (hereafter ADP1) has been investigated and includes several analogous substrates, no activity for syringate has been previously detected [36]. This is unexpected given the high similarity between *vanAB* orthologues in the different strains. Many factors support the hypothesis of syringate *O-*demethylation by VanAB in ADP1. For example, the amino acid sequence identity of the substrate binding VanA found in ADP1 to *P. putida* KT2440 and *P. putida* HR199 is very high, 75–78% (Supplementary Table S1-3, Additional file 1). Known substrates of VanAB indicate that a methoxy group in meta position to a carboxyl group is necessary for the activity of the enzyme [36]. This does not exclude syringate as a substrate.

ADP1 has raised increasing interest as a potential microbial cell factory for its versatile metabolism and natural competence, which make metabolic engineering remarkably simple [37–39]. ADP1 can utilize a variety of G- and H-type lignin monomers via the native β-ketoadipate pathway and has been shown to have ligninolytic effects on softwood [40, 41]. Previously, ADP1 has been engineered for the production of wax esters and alkanes [42], 1-alkenes [43], *cis*,* cis*-muconate [44], mevalonate [45], naringenin [46], and resveratrol and vanillin-glucoside [47] from lignin-related aromatic compounds, such as ferulate and *p*-coumarate. In addition, the strain has been previously evolved to tolerate very high concentrations of aromatic compounds [43, 48] as well as exploited in the detoxification of lignocellulosic hydrolysates [49–52].

In this study, our goal was to establish the conversion and upgrading of S-lignin-related syringate in ADP1. We first investigated the activity of VanAB both in vivo and in vitro under native and non-native expression systems. We showed that both syringate and 3MGA can be *O-*demethylated by ADP1. We also demonstrated the production of PDC, a precursor for high-quality polyesters, from syringate by the heterologous expression of gallate dioxygenase GalA from *P. putida* KT2440.

## Materials and methods

### Strains and media

*Escherichia coli* XL1-Blue (Stratagene, USA) was used for plasmid construction and maintenance. *Acinetobacter baylyi* ADP1 (DSM 24193, Leibniz Institute DSMZ, Germany) was used to study the endogenous expression of *vanAB* and for strain construction. All the strains used and engineered in this study are listed in Table 1.

**Table 1:** Bacterial strains used in this study

Name	Genotype	Description		Source	
ADP1 WT	Wild-type *A. baylyi* ADP1	DSM 24193		DSMZ	
ASA1001	*A. baylyi* ADP1 Δ*vanAB*::*tdk/kan*^*r*^	*vanAB* (ACIAD0979-0980) replaced with *tdk/kan*^*r*^ cassette		This study	
ASA1002	*A. baylyi* ADP1 Δ*vanAB*	Strain with markerless *vanAB* deletion		This study	
ASA1003	*A. baylyi* ADP1 Δ*vanAB*, harboring pBAV1C-T5-*vanAB*	Strain ASA1002 with *vanAB* overexpression from pBAV1C-T5 plasmid under constitutive T5 promoter.		This study	
ASA1004	*A. baylyi* ADP1 Δ*vanAB*, harboring pBAV1Cd-chn-*vanAB*	Strain ASA1002 with *vanAB* overexpression from pBAV1Cd-plasmid under cyclohexanone- inducible ChnR/P_*chnB*_ promoter. N-terminal His-tag in *vanA* for purification.		This study	
ASA1005	*A. baylyi* ADP1 harboring pKLxR5- *galA* (Gm)	Strain with plasmid *galA* overexpressionfrom pKLxR5 plasmid under AHL-inducible LuxR/P_*luxB*_ promoter.		This study	
**ASA902**	*A. baylyi* ADP1 Δ*pcaHG*	Strain with markerless *pcaHG* (ACIAD1711-1712) deletion		[53]	
*Pseudomonas (allo)putida* KT2440	Wild-type	DSM 6125, natively harbors *galA* gallate dioxygenase (PP_2518, *glllA*)		DSMZ	

Modified low salt lysogeny broth (LB) medium (10 g/L tryptone, 5 g/L yeast extract, 1 g/L NaCl) supplemented with 50 mM glucose was used to cultivate *E. coli* and ADP1 for strain construction. Mineral salts medium (MSM) supplemented with 50 mM (unless stated otherwise) glucose, 0.2% casein amino acids, and aromatics in specified concentrations was used in aromatics conversion experiments. MSM composition was 3.88 g/L K_2_HPO4, 1.63 g/L NaH_2_PO_4_, 2.00 g/L (NH_4_)_2_SO_4_, 0.1 g/L MgCl_2_·6 H2O, 10 mg/L ethylenediaminetetraacetic acid (EDTA), 2 mg/L ZnSO_4_·7 H2O, 1 mg/L CaCl_2_·2 H_2_O, 5 mg/L FeSO_4_·7 H_2_O, 0.2 mg/L Na_2_MoO_4_·2 H_2_O, 0.2 mg/L CuSO_4_·5 H_2_O, 0.4 mg/L CoCl_2_·6 H_2_O, and 1 mg/L MnCl_2_·2 H_2_O. Antibiotics for *E. coli* and ADP1 were supplemented when needed in following concentrations: chloramphenicol, 25 µg/ml; gentamicin, 15 µg/ml; spectinomycin, 50 µg/ml; kanamycin 30 µg/ml. Zidovudine plates in concentration 400 µg/ml were prepared for counter selection of the gene deletions. N-(3-oxohexanoyl) homoserine lactone (AHL) and cyclohexanone were used for induction in specified concentrations ranging from 1 to 10 µM.

Aromatics stocks were prepared in following concentrations: vanillate 200 mM, syringate, 3MGA, and gallate 100 mM. Correct amount of the aromatic compound was weighed and added to deionized water after which KOH was slowly added to equimolar or slightly excess amount to dissolve the compounds as potassium salts, with final pH reaching 8.2–8.5. Reagents were purchased from Sigma Aldrich (United States).

### Genetic engineering

Genetic engineering was carried out using established methods for restriction-ligation, Uracil-Specific Excision Reagent (USER) and NEBuilder^®^ HiFi DNA Assembly cloning as well as overlap extension PCR. Reagents for molecular work were purchased from Thermo Scientific (USA) and New England Biolabs (USA) and used according to the manufacturer’s instruction. The primers used in this study are listed in Supplementary Table S4 (Additional File 1). Electroporation was used for the transformation of *E. coli* XL-1 Blue. ADP1 transformation and genomic editing by homologous recombination were carried out as described previously by [54]. The transformants were screened on lysogeny agar (LA) plates with appropriate antibiotics.

*VanAB* was deleted from ADP1 genome using a linear DNA cassette; regions of approximately 1 kb flanking *vanAB* to facilitate homologous recombination were amplified from ADP1 genome with primers *VanB_P5-OE* and *VanB R2* (3’ segment), and *VanA_P4-OE* and *VanA F2* (5’ segment). The fragment *tdk/kan*^*r*^ carrying kanamycin resistance marker and *tdk* for counter-selection [55] was amplified with primers Tdk_kanF and Tdk_kanR from the genome of ADP1∆3383::*tdk/kan*^*r*^, (a kind gift from Dr. Veronique de Berardinis, Genoscope, France) [56]. The cassette was assembled with overlap extension PCR and transformed into ADP1 WT resulting in strain ASA1001. Kanamycin was used for selection of successful clones. A rescue cassette to replace *tdk/kan*^*r*^ was constructed in similar manner; flanking regions of *vanAB* were amplified with primers *rescue cassette vanB forward* and *vanB R2* (3’ flanking) and *vanA F2* and *rescue cassette vanA reverse* (5’ flanking). Restriction sites for MfeI, NotI and AvrII were designed into overlapping primers *rescue cassette vanB forward* and *rescue cassette vanA reverse* that join the flanking sequences together (Supplementary Table S4, Additional File 1). Overlap extension PCR was used to combine the segments.

The rescue cassette was transformed into ASA1001 resulting in ASA1002 with markerless *vanAB* deletion. Counterselection with zidovudine was used to select successful clones; After transformation with the rescue cassette, the cell culture was diluted 1:10, 1:100 and 100 µl volumes were spread on LA plates supplemented with 400 µg/ml zidovudine and 50 mM glucose. The plates were incubated at 30 °C until colonies appeared. Colonies were then resuspended in LB media and divided into two cultures each, in LB media supplemented with 50 mM glucose and either 30 µg/ml kanamycin or no antibiotic. The cultures were incubated at 30 °C, 300 rpm overnight, after which those that did not grow on kanamycin were selected. The integration of the rescue cassette was further confirmed with PCR (Supplementary Figure S1, Additional file 1).

Plasmid pBAV1Cd-chn-*vanAB* for overexpression of *vanAB* was constructed by BioBrick cloning in the empty plasmid pBAV1Cd-chn [43]. To clone *vanAB* from ADP1 genome it was amplified with primers *VanAB_F_BB* and *VanAB_R_BB.* A ribosome binding site, a new start codon, His (6)-tag and Gly-Ser-Gly -linker sequence were included in the forward primer *VanAB_F_BB* annealing to *vanA* to enable purification with the histidine tag (Supplementary Table S4, Additional File 1). The obtained plasmid pBAV1Cd-chn-*vanAB* was first transformed into *E. coli* XL1-Blue and then into ASA1002 resulting in ADP1Δ*vanAB* pBAV1Cd-chn-*vanAB* (ASA1004).

Next, *vanAB* was cloned under a constitutive T5-promoter with USER cloning. *VanAB* from ADP1 genome was amplified with primers SS-21-03-IFU and SS-21-04-IRU. SS-21-01-VFU and SS-21-02-VRU were used to amplify the backbone of pBAV1C-T5-GFP [57]. The obtained plasmid pBAV1C-T5-*vanAB* was first transformed into *E. coli* XL1-Blue and then into ASA1002 resulting in ADP1ΔvanAB pBAV1C-T5-*vanAB* (ASA1003).

To overexpress *galA*, plasmid pKLxR5-*galA* was constructed with NEB HiFi assembly. *GalA* was amplified from *P. putida* KT2440 genome with primers *GalA_F* and *GalA_rev*. Plasmid pKLxR5-mRFP, a kind gift from Schuster and Reisch, Addgene #149465 [58] was utilized as a backbone and amplified with primers *pKLxR5_fwd* and *prR*. The obtained plasmid pKLxR5-*galA* was transformed into *E. coli* XL1-Blue and then into ADP1 WT resulting in strain ASA1005.

### Growth experiments

#### Characterization of syringate tolerance and growth on vanillate on microplates

Effects of *vanAB* deletion and overexpression were characterized by cultivating the strains on vanillate as a sole carbon source. Overnight precultures were prepared in 5 ml volume of MSM, 0.2% casein amino acids, and 25 µg/ml chloramphenicol (dissolved in MQ) when appropriate. The following day, the precultures were used to inoculate culture medium containing MSM, 5 mM vanillate, 25 µg/ml chloramphenicol when appropriate, and 5 µM cyclohexanone for the induction of ASA1004. Each strain was cultivated in triplicates on 96-well plate (200 µl medium/well). The plates were incubated in Spark multimode microplate reader (Tecan, Switzerland) at 30 °C. The cultures were mixed with double orbital shaking twice per hour with an amplitude of 6 mm and frequency of 54 rpm. Optical density at 600 nm was measured twice per hour.

For studying the syringate tolerance, strains ADP1 WT, ASA1002, ASA1003 and ASA1004 were precultured overnight in 5 ml volume in MSM media with 0.2% casein amino acids, 20 mM glucose, and appropriate antibiotics. Each strain was cultivated in duplicates on a 96-well plate (200 µl medium/well) in the same media as the precultures supplemented with 50 mM glucose and 0, 1, 2, 5, 10, and 20 mM syringate. The plates were incubated in Spark multimode microplate reader (Tecan, Switzerland) as described above.

#### Batch cultivations for syringate conversion and production of PDC

Overnight precultures were inoculated from single colonies on LA plates. Precultures were carried out in 5 ml MSM supplemented with 0.2% casein amino acids, 50 mM glucose, and appropriate antibiotics. The main cultivations were carried out in 15 ml MSM media in 50 ml Nunc™ bioreactor tubes (Thermo Scientific, United States).

Culture media was supplemented with 0.2% casein amino acids, 50 mM glucose, appropriate antibiotics, and 5 mM syringate with a starting OD of 0.1–0.2. Cyclohexanone was added to the cultivations in concentrations 0–5 µM to induce *vanAB* expression in ASA1004. Induced cultivations were kept on separate incubator from uninduced ones to avoid any effect of the highly volatile cyclohexanone. The pH of the media was monitored in the beginning and at the end of the cultures with pH strips.

For quantification of metabolites, 1 ml samples were collected and centrifuged at 14 000 g for 2 min. The supernatants were collected and stored at -20 °C prior to analysis. The samples were diluted in deionized water to appropriate concentration (maximum 10-fold dilution) and filtered with 0.2 μm filters before analysis. The pellets were washed with and resuspended to MSM after which OD600 was measured because of the dark coloration formed during the cultivations.

Cultivation for PDC production was carried out similarly. Precultures were continued overnight, after which appropriate volumes were used for inoculation to achieve initial OD of 0.1–0.2. 1 µM AHL was added to strain with *galA* under LuxR-regulated promoter for induction. *VanAB* expression was not induced.

#### Fed-batch cultivations for production of PDC

Bioreactor experiments for PDC production were carried out in small scale bioreactors (Applikon Biotechnology, Netherlands) with up to 250 ml working volume. The reactor was maintained at 30 °C, 200 rpm and at a constant aeration. Dissolved oxygen and pH were monitored during the cultivations. Media pH was adjusted to 6.0–7.0 with potassium hydroxide. When necessary, 10% antifoam A (Fluka Analytical) was added. Samples of 1–2 ml for analysis of OD600 and aromatics were collected and handled as described previously in batch cultivations. Starting volume of the cultivations was 50 ml of MSM media supplemented with 10 mM glucose, 0.2% casein amino acids, 10 µM AHL, 15 µg/ml gentamicin, syringate and vanillate in specified concentrations, and inoculant resulting in initial OD of approximately 4. Feed containing 75 mM glucose and syringate and vanillate in specified concentrations was pumped to the reactor at 3.0-3.6 ml per hour. The feed contained other components in same concentrations as in the initial media. Two independent biological replicates were produced by individual experiments.

ASA1005 was inoculated in 5 ml of MSM supplemented with 50 mM glucose, 0.2% casein amino acids, and 15 µg/ml gentamicin at 30 °C, 300 rpm. After overnight cultivation, the cells were inoculated in the second preculture media in 15 ml of MSM supplemented with 50 mM glucose, 0.2% casein amino acids, 15 µg/ml gentamicin, and 10 µM AHL to induce *galA* expression, as well as 1 mM vanillate to induce native *vanAB* expression, with initial OD 0.1–0.2. The cells were cultivated overnight in 50 ml bioreactor tubes in RTS-8 multichannel bioreactor (Biosan, Latvia) at 2700 rpm, 3 s^− 1^ RSI (reverse spin interval), 30 °C. Parallel cultivations were pelleted at 5000 g for 10 min and resuspended in 15 ml MSM to achieve initial OD of 4 in bioreactor after inoculation.

### Analytical methods

Gallic acid (GA) was purchased from Merck (Switzerland), 3,4-dihydroxy-5-methoxybenzoic acid (3MGA), syringic acid (SA), vanillic acid (VA) and protocatechuic acid (PCA) were purchased from Sigma (USA). Methanol (HPLC grade) was purchased from Honeywell (Germany). All standards of aromatic compounds were prepared as 20 mM stock solutions in water by adding of 5 M KOH until full solubilization as described above. For calibration, working standard solutions were prepared by diluting of stock solutions with water to concentrations 0.5–5 mM and filtered using 0.2 μm filters. HPLC analysis of the aromatic compounds and PDC was performed on Shimadzu LC-40 (Japan), equipped with a photodiode array detector (PDA). The compounds were analysed on the column Rezex RFQ-Fast Acid H+ (8%), 100 × 7.8 mm, 55 °C (Phenomenex Inc., USA) in 5 mM H_2_SO_4_ at the flow rate of 0.6 mL/min. The injection volume was 5 µL. Eluted GA, 3MGA and SA were monitored at wavelength 272 nm. VA and PCA eluted at 22.3 min and 11.2 min, respectively, were monitored at wavelength 254 nm.

Since a commercial PDC standard is not available, we used a sample collected from a cultivation of ADP1 containing PDC as a qualitative standard. The presence of PDC in the qualitative standard was proven by mass-spectrometry and nuclear magnetic resonance (^1^H-NMR). Mass-spectrometric analysis was carried out using JEOL AccuTOF LCplus (JMS-T100LP) (Japan) in ESI- mode. The molecular ion of PDC was detected as [M-H]^−^ 182.96624 and distinguished from the molecular ion of 3-*O*-methyl gallate [M-H]^−^ 183.02990, the compound with almost equal molecular weight. As an additional proof, the ^1^H-NMR spectra of the qualitative standard and 3-*O*-methyl gallate were recorded on a JEOL spectrometer ECZ500R 500 MHz (Japan) in D_2_O. Characteristic signals of the two protons of ɑ-pyrone ring at 6.74 (s) and 7.23 (s) ppm were detected in the spectrum of the tested sample whereas characteristic signals of aromatic protons and methoxy protons of 3-*O*-methyl gallate (7.02, 7.07 and 3.78, respectively) were not found in the spectrum. Quantification of PDC was performed from its chromatographic peak area using extinction coefficient e = 6200 M^− 1^ .cm^− 1^ at 313 nm reported by Michinobu et al. [59].

### VanAB production and purification

For VanAB production, 10 ml overnight culture of ASA1004 was grown at 30 °C in low salt LB medium supplemented with 50 mM glucose and 25 µg/ml chloramphenicol. The following day, cells were diluted in 100 ml of fresh medium (supplemented also with 20 µM FeSO_4_) to initial OD_600_ of 0.05, and grown at 30 °C. When OD_600_ reached 0.5–0.8, cyclohexanone was added as an inducer of *vanAB* expression to a final concentration of 5 µM. Cultivation was continued at 25 °C for approximately 20 h. Cells were harvested by centrifugation at 30 000 *g* for 30 min at 4 °C and stored at -20 °C.

For the copurification of VanAB, the cell pellets were thawed and resuspended in BugBuster Protein Extraction Reagent (Novagen, USA) using 5 ml reagent per gram of wet cell paste, or more if necessary. Lysozyme was then added to a final concentration of 1–2 mg/ml and incubated while gently mixing for 30 min at room temperature. Disruption by sonication was performed in sequence of 15 s sonication followed by a 15 s pause on Fisherbrand™ Model 120 Sonic Dismembrator (Thermo Fisher Scientific, USA) while kept on ice. The cell lysate was cleared by centrifugation at 30 000 *g* for 20 min at 4 °C. Imidazole was added to the cleared lysate to a final concentration of 20 mM. The cleared lysate was loaded on to a HisGraviTrap Ni-Sepharose column (GE, USA) and washed with binding/washing buffer (20 mM Tris, 500 mM NaCl, 20 mM imidazole, pH 8). VanAB was eluted with elution buffer (20 mM Tris, 500 mM NaCl, 500 mM imidazole, pH 8) and dithiothreitol (DTT) was added to a final concentration of 5 mM to increase the stability of the protein. Purity of the protein preparation was estimated by Sodium Dodecyl Sulfate Polyacrylamide Gel Electrophoresis (SDS-PAGE) based on the method described by Laemmli [60] using a 12% precast polyacrylamide gel (Bio-Rad, USA). An image of a typical SDS-PAGE gel from VanAB co-purification is shown in Supplementary Figure S2, Additional File 1.

### VanAB activity assay

Activity assay for the purified VanAB was performed the following day due to loss of activity after longer storage. Prior to assay, PD MidiTrap G-25 (Cytiva, USA) column was used for buffer exchange into 20 mM Tris-HCl, 100 mM NaCl, 5mM DTT, pH 8. The activity assay was based on monitoring the consumption of NADH by measuring the absorbance at 340 nm. Reactions were performed at 25 °C with a range of substrate concentrations in 20 mM Tris-HCl, 100 mM NaCl, 5 mM DTT, pH 8. When vanillate or syringate was used as a substrate, two-fold successive dilution series were prepared to give final concentrations in reactions ranging from 400 µM to 25 µM. For 3MGA, substrate concentrations in reactions starting from 3200 µM to 200 µM were used. Control reactions were performed with 400 µM PCA and without any substrate. All reactions contained initial 400 µM NADH. Reactions were initiated by the addition of 40 µl purified VanAB preparation into total reaction volume of 200 µl. The absorbance at 340 nm was measured with Spark multimode microplate reader (Tecan, Switzerland) every 3 min until 90 min in total. The oxidation of NADH was converted to the amount of consumed NADH. Initial velocities, *v*_*1*_, at different substrate concentrations, [S] were fitted to the Michaelis-Menten equation shown in Eq. 1

1 $$ v_{i} = \frac{{V_{{max}} ~ \cdot \left[ S \right]}}{{K_{M} + \left[ S \right]}} $$

Where *K*_M_ is the Michaelis-Menten constant and *V*_max_ is the maximum velocity of the reaction.

## Results

### Characterization of the growth and conversion of vanillate and syringate by VanAB expressing strains

**Fig. 2:**
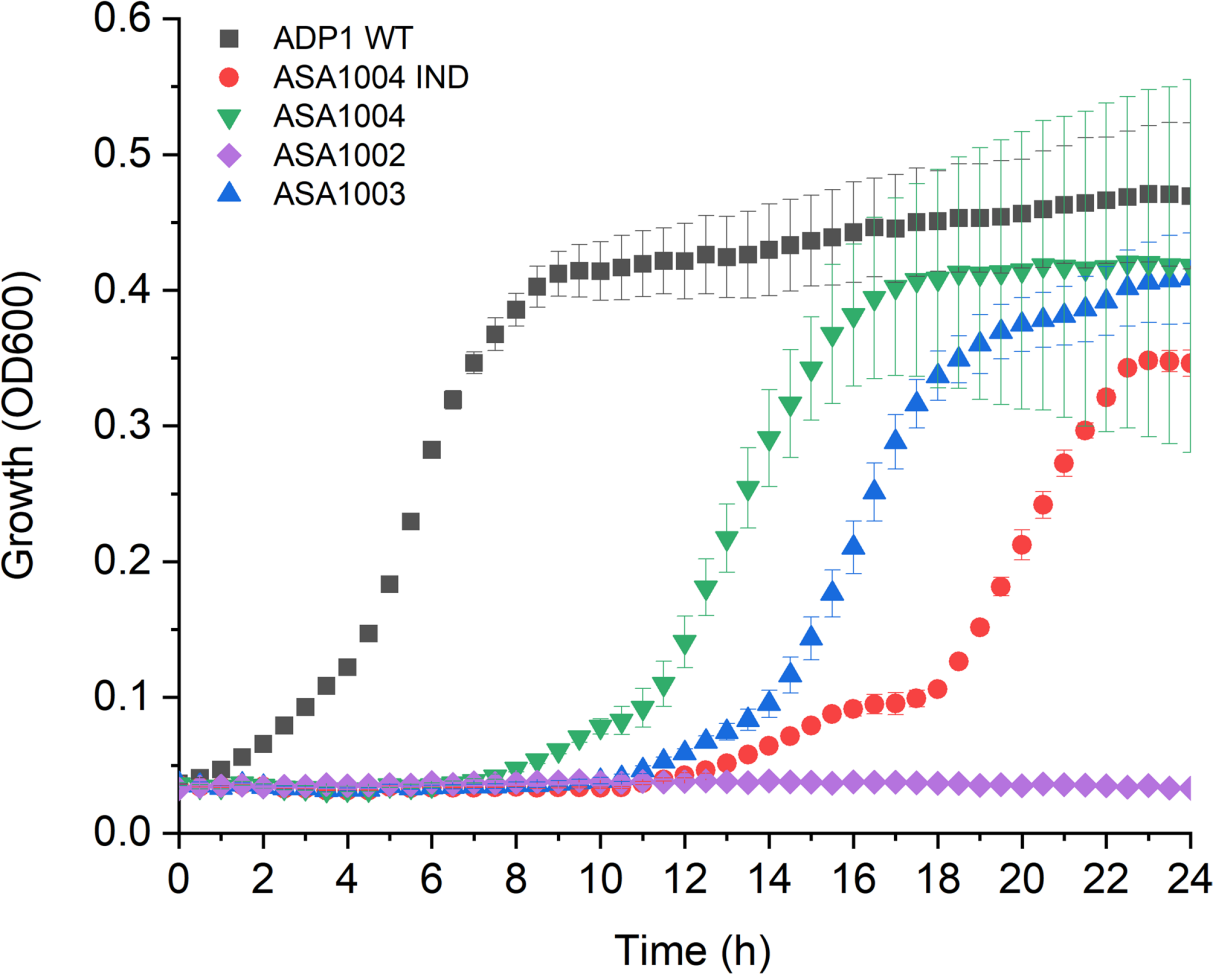
The growth of ADP1 WT, ASA1002, ASA1003, induced ASA1004 IND (5 µM cyclohexanone), and uninduced ASA1004 on vanillate as a sole carbon source. The strains were cultivated in MSM supplemented with 5 mM vanillate and 25 µg/ml chloramphenicol for ASA1004. The strains were cultivated in 200 µl on a 96 well plate at 30 °C with shaking for 24 h. The mean values and error bars representing the standard deviations from three parallel cultures are shown

We first investigated the *O-*demethylation activity of ADP1 VanAB by native and non-native expression systems. First, the native copy of *vanAB* was deleted from the genome resulting in strain ASA1002. Two plasmid-based overexpression systems, pBAV1C-T5-*vanAB* and pBAV1Cd-chn-*vanAB*, were transformed into ASA1002, resulting in strains ASA1003 and ASA1004 with constitutive and cyclohexanone-inducible *vanAB* expressions, respectively. The strains were cultivated with vanillate, the known substrate for VanAB, as the sole carbon source (Fig. 2). Overexpression of *vanAB* resulted in nearly 10-hour lag phase, while for ADP1 WT noticeable lag phase was not observed. Interestingly, for the uninduced strain, the lag-phase was slightly shorter compared to overexpressing strains. A low expression level of *vanAB* can be expected due to leakiness of the *ChnR*/P_*chnB*_ [61]. As expected, ASA1002 with *vanAB* deletion did not grow on vanillate.

**Fig. 3:**
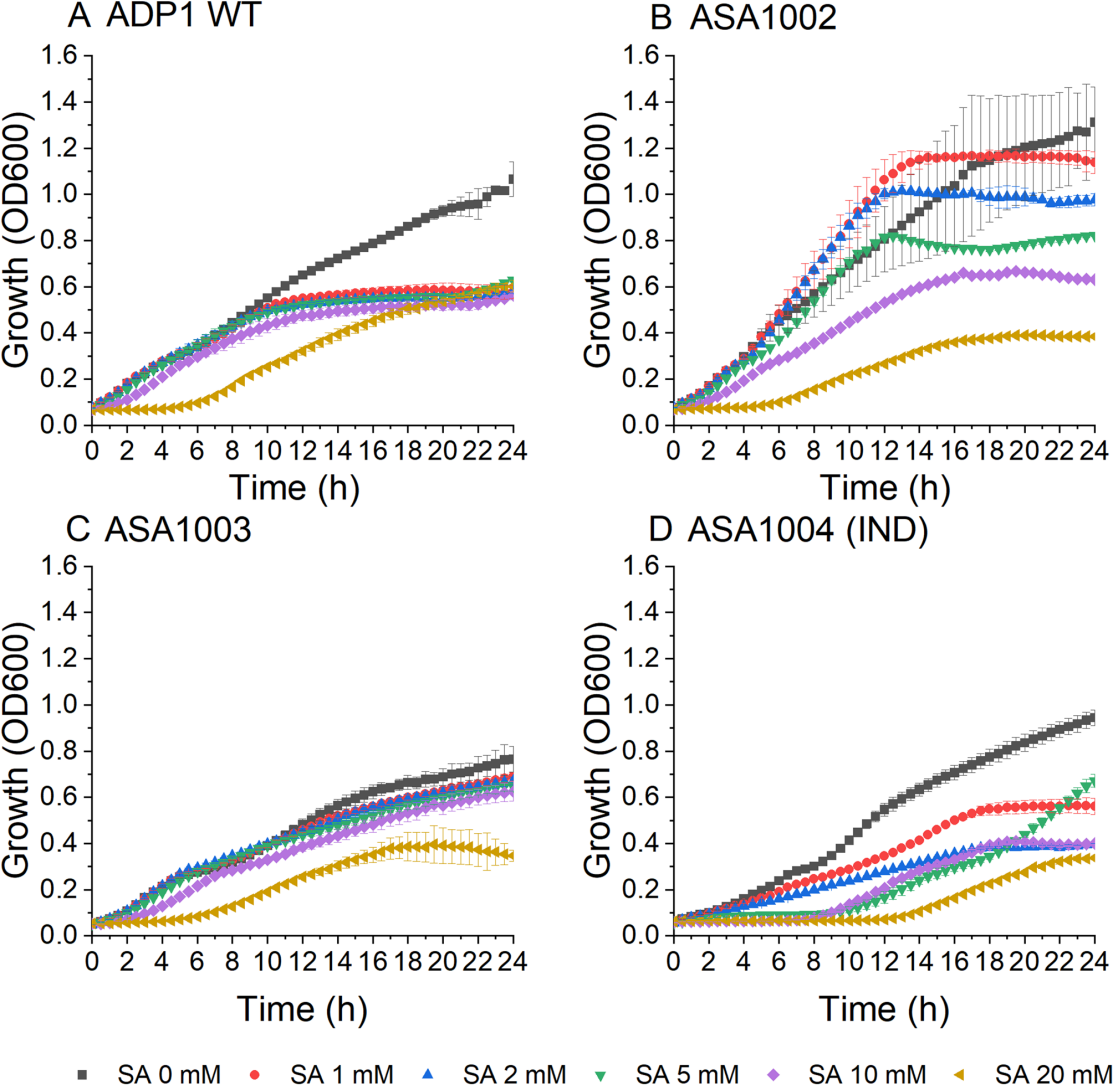
Syringate tolerance of A: ADP1 WT, B: ASA1002, C: ASA1003 and D: ASA1004 (induced with 5 µM cyclohexanone). The strains were cultivated in mineral salts medium supplemented with 0–20 mM syringate, 50 mM glucose, 0.2% casein amino acids, and 25 µg/ml chloramphenicol where required. The strains were cultivated in 200 µl media on a 96 well plate for 24 h. The mean values and error bars representing the standard deviations from two parallel cultures are shown. SA: syringate

Next, the syringate tolerance of ADP1 WT and the engineered strains was determined (Fig. 3). The media was supplemented with 50 mM glucose and syringate in concentrations ranging from 0 to 20 mM. At the highest concentration of syringate tested (20 mM), strains ADP1 WT, ASA1002, and ASA1003 had approximately a 6-hour lag phase, whereas the induced ASA1004 strain had even a longer lag phase, approximately 12 h. In all strains, syringate negatively impacted growth in comparison to cultivations with only glucose, and the growth of induced ASA1004 was hindered even at low syringate concentrations. By contrast, for ASA1002, only the highest concentrations of syringate resulted in clearly reduced overall growth. Close to the end of the cultivation, a medium color change from clear to black was noticed in cultures of ADP1 WT and ASA1003 (Supplementary Figure S3, Additional File 1). To eliminate any potential influence of the medium color change on growth assessment, we evaluated the syringate tolerance of ADP1 WT, ASA1002, and ASA1004 also in larger volume cultures. Prior to OD measurement, samples were washed to ensure accuracy (Supplementary Figure S4, Additional File 1).

### Syringate and 3MGA conversion by VanAB in wild-type and engineered ADP1

**Fig. 4:**
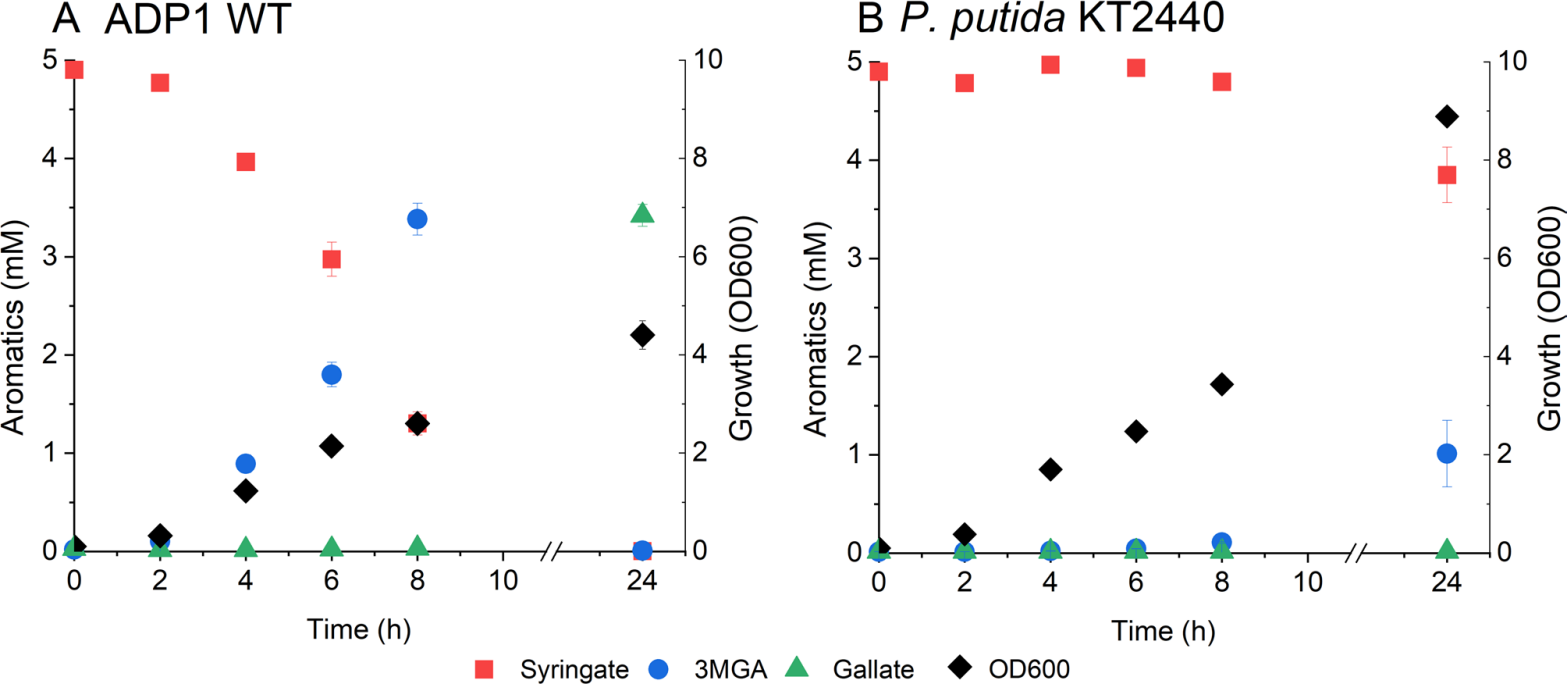
O-demethylation of syringate by A: ADP1 WT, B: *P. putida * KT2440. The strains were cultivated in 15 ml mineral salts medium supplemented with 5 mM syringate, 50 mM glucose, and 0.2% casein amino acids. Samples were collected at the indicated timepoints to monitor growth (OD600) and metabolite concentrations in the media (analysed with HPLC). The mean values and error bars representing the standard deviations from two parallel cultures are shown. 3MGA: 3-O-methylgallate

Based on the tolerance test, 5 mM concentration was selected for studying syringate conversion in ADP1 in vivo. We first tested the conversion in ADP1 WT and compared the performance to that of *P. putida* KT2440, which has been previously shown to *O-*demethylate syringate in the presence of an auxiliary carbon source, resulting in the accumulation of 3MGA [30]. The strains were cultivated in MSM supplemented with 50 mM glucose, 0.2% casein amino acids, and 5 mM syringate (Fig. 4). Both syringate and 3MGA were completely *O-*demethylated by ADP1 WT within 24 h. Only small amount of syringate was converted to 3MGA by *P. putida*, being in line with previous research [30].

**Fig. 5:**
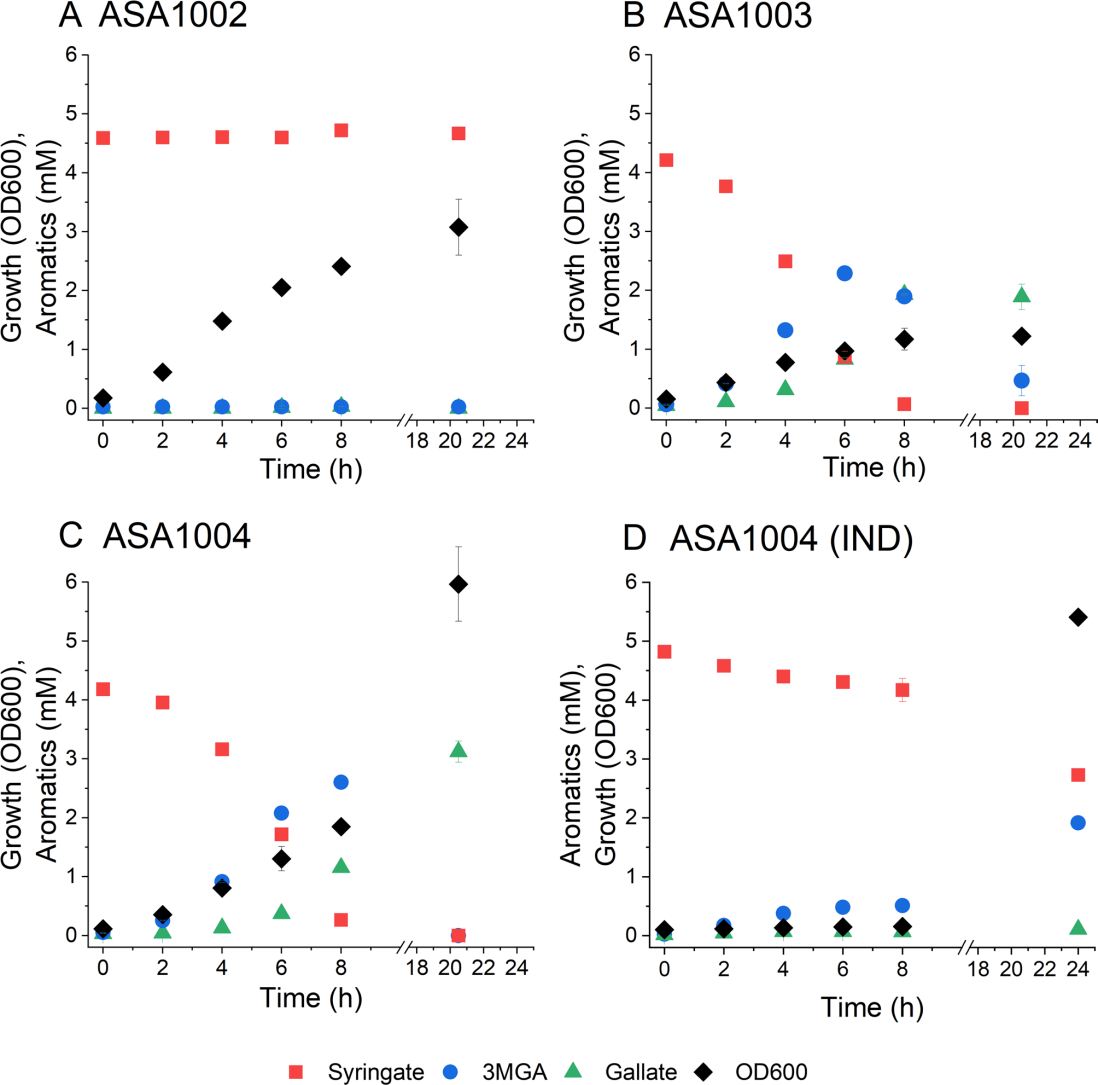
O-demethylation of syringate by A: ASA1002 B: ASA1003, C: ASA1004, D: ASA1004 induced with 5 µM cyclohexanone. The strains were cultivated in 15 ml mineral salts medium supplemented with 5 mM syringate, 50 mM glucose, 0.2% casein amino acids, and 25 µg/ml chloramphenicol when required. Samples were collected at the indicated timepoints to monitor growth (OD600) and metabolite concentrations in the media (analysed with HPLC). The mean values and error bars representing the standard deviations from two parallel cultures are shown. Abbreviations: 3MGA: 3-O-methylgallate

We then investigated the syringate conversion by ASA1003 and ASA1004. ASA1002 was used as the control. The strains were cultivated in MSM supplemented with 50 mM glucose, 0.2% casein amino acids, and 5 mM syringate and appropriate antibiotics (Fig. 5). Interestingly, the strains ASA1003 and ASA1004, which overexpress *vanAB*, exhibited different reaction dynamics compared to ADP1 WT. In these strains, syringate and 3MGA were *O-*demethylated simultaneously, unlike in ADP1 WT, where syringate was depleted first. Notably, the induction of ASA1004 caused an over 8-hour lag phase, resulting in much slower overall conversion of syringate. A very minor decrease in syringate concentration was observed in cultivations with ASA1002, likely due to abiotic degradation. These results indicate that VanAB is indeed responsible for syringate conversion.

To screen for the optimal induction level in ASA1004, different cyclohexanone concentrations up to 5 µM were tested (Supplementary Figure S5, Additional File 1). We found that very low expression levels of *vanAB*, even without induction, are sufficient for the conversion, whereas too high expression almost completely inhibits growth (Supplementary Figure S6, Additional File 1). Again, dark coloration was observed after approximately 24 h of cultivation most likely caused by the abiotic oxidation of gallate, which results in a dark gray or black coloration and precipitate [7, 62–64].

To confirm that the *O-*demethylation of 3MGA is also carried out by VanAB, we cultivated ADP1 WT and ASA1002 in media supplemented with 3MGA instead of syringate (Supplementary Figure S7, Additional File 1). As expected, ADP1 WT promptly *O-*demethylated 3MGA to gallate, while no conversion above the abiotic rate was detected for ASA1002. To prospect for the possibility of unknown enzymes capable of syringate conversion or further syringate metabolism that could enable growth, ADP1 WT, ASA1002, and ASA1004 were cultivated with syringate as the sole carbon source (Supplementary Figure S8, Additional File 1). Minor conversion of syringate into 3MGA was observed, but neither further conversion to gallate nor growth was detected. The findings indicate that syringate and 3MGA *O-*demethylation in ADP1 is exclusively carried out by VanAB.

To investigate potential unknown metabolic activities leading to gallate degradation, we cultivated ADP1 WT and ASA1005 in MSM media supplemented with casein amino acids and either 50 mM glucose, 5 mM gallate, or both (Supplementary Figure S9, Additional file 1). The experiment revealed a rapid decrease in gallate concentration even under abiotic conditions. The presence of casein amino acids, glucose, or their combination did not affect the consumption of gallate. In fact, gallate was observed to inhibit the growth of ADP1. Thus, it was concluded that gallate is not further catabolized to central metabolites in ADP1.

### VanAB activity in vitro

**Fig. 6:**
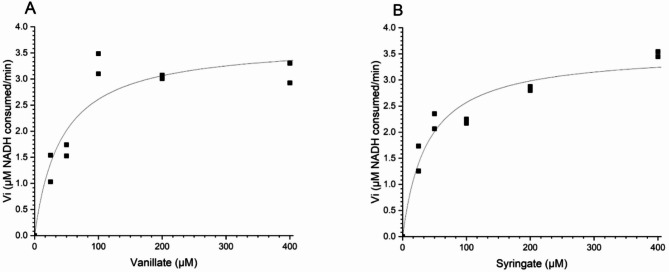
The saturation curves for VanAB with A: vanillate and B: syringate as a substrate. The curves are generated from fitting Eq. 1 of v_1_ of consumed NADH versus [S]. Experiments were performed as described in Materials and methods. Duplicate reactions at each substrate concentration were performed

To investigate the substrate preference of VanAB, the strain ASA1004 was cultivated for the production of VanAB which were co-purified for enzymatic activity assay. Notably, the overexpression of *vanAB* in the absence of a specific substrate for VanAB did not negatively impact the growth of ASA1004. The purified protein preparation had a red-brown color (Supplementary Figure S2B, Additional File 1), which is characteristic of Rieske-type oxygenases such as VanA [65]. The activity of VanAB towards different substrates was assayed based on monitoring the consumption of NADH. The results indicated a clear coupling of NADH consumption by VanAB in the presence of vanillate or syringate in the reaction, as opposed to the minimal consumption of NADH in the presence of PCA or without substrate (Supplementary Figure S10, Additional File 1). The saturation curves for VanAB with vanillate and syringate as a substrate are shown in Fig. 6. Using vanillate and syringate as substrates, the Michaelis-Menten constants (*K*_M_) were found to be 42 ± 15 µM and 38 ± 9 µM, respectively. These results align with previous studies on homologous enzyme from *P. putida* KT2440 [30]. Interestingly, syringate appears to be as good a substrate as vanillate for the VanAB of ADP1. However, we were unable to detect in vitro activity when 3MGA was used as a substrate under the studied conditions, presumably because of the much lower catalytic ability of VanAB with 3MGA. Nevertheless, VanAB-mediated *O-*demethylation of 3MGA was observed in the in vivo cultivations (Fig. 4 and Supplementary Figure S7, Additional File 1).

### Production of PDC in ADP1

**Fig. 7:**
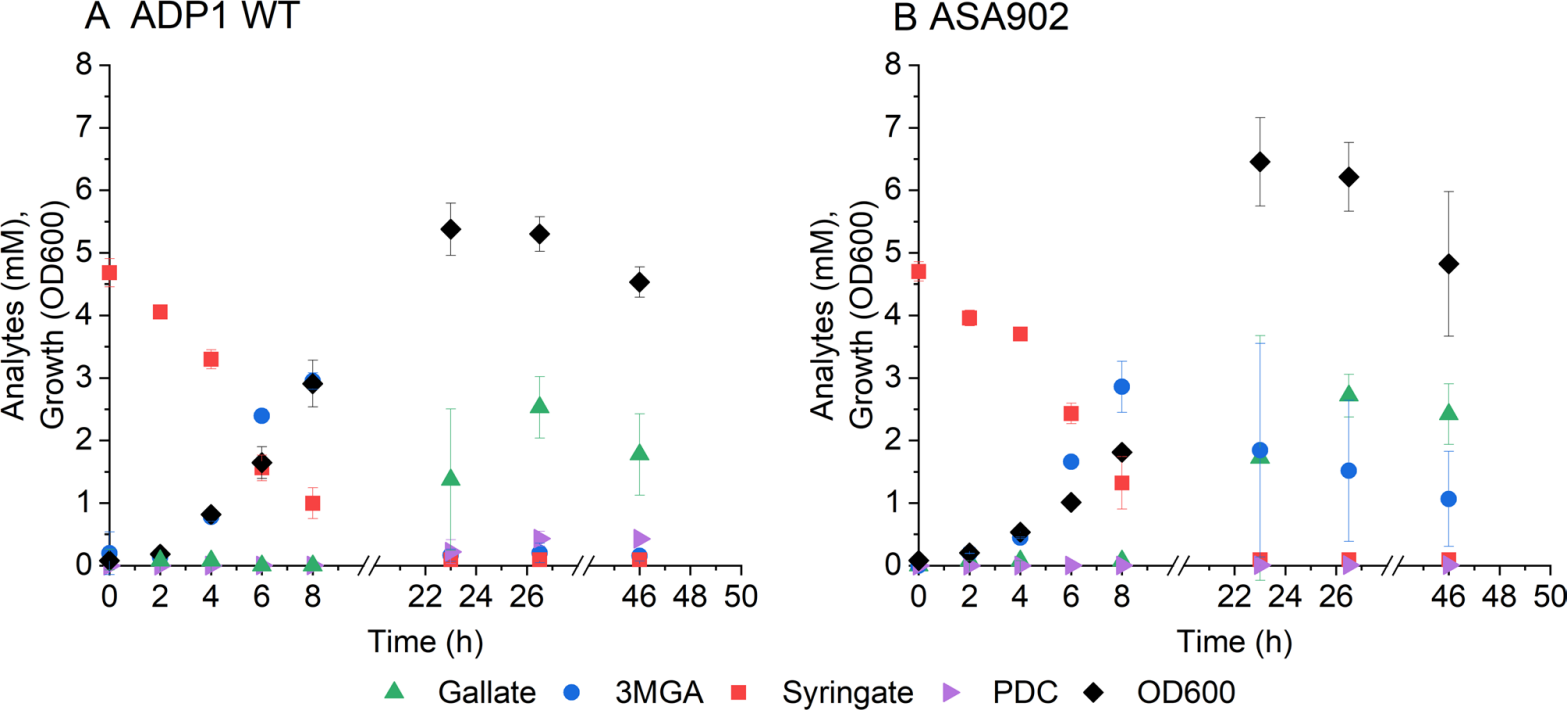
Syringate conversion into PDC by A: ADP1 WT B: ASA902. The strains were cultivated in 15 ml mineral salts medium supplemented with 5 mM syringate, 50 mM glucose, and 0.2% casein amino acids. Samples were collected at the indicated timepoints to monitor growth (OD600) and metabolite concentrations in the media (analysed with HPLC). The mean values and error bars representing the standard deviations from three parallel cultures are shown. 3MGA: 3-O-methylgallate; PDC: 2-pyrone-4,6-dicarboxylate

The robust conversion of syringate by VanAB encouraged us to further explore potential production pathways. PDC is an exciting product of aromatic catabolism, as several lignin-derived aromatics can be simultaneously funnelled towards its production. In the VanAB-mediated pathway, there are two known routes to PDC from syringate: through 3MGA and gallate [30]. Protocatechuate dioxygenase PcaHG of *P. putida* KT2440 has been reported to convert gallate into PDC [30]. To test the corresponding activity of the homologous PcaHG in ADP1, we first cultivated ADP1 WT and a control strain ASA902 with *pcaHG* deletion in media supplemented with 5 mM syringate and 50 mM glucose. Small amounts of PDC were detected from ADP1 WT cultures (Fig. 7), indicating potential, albeit minor activity of PcaHG towards gallate.

**Fig. 8:**
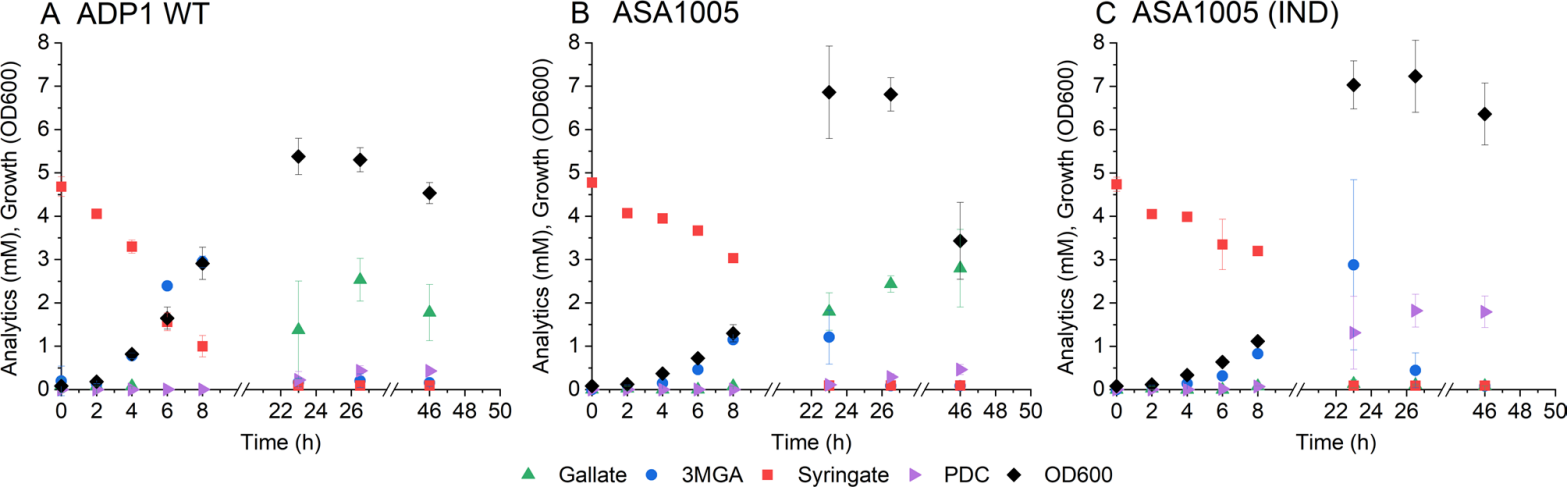
Syringate conversion into PDC by A: ADP1 WT, B: ASA1005 C: ASA1005 induced with 1 µM AHL. The strains were cultivated in 15 ml mineral salts media supplemented with 5 mM syringate, 50 mM glucose, and 0.2% casein amino acids. In addition, for ASA1005 15 µg/ml gentamicin and 1 µM AHL for the induction of galA were supplemented. Samples were collected at the indicated timepoints to monitor growth (OD600) and metabolite concentrations in the media (analysed with HPLC). The mean values and error bars representing the standard deviations from three parallel cultures are shown. 3MGA: 3-O-methylgallate; PDC: 2-pyrone-4,6-dicarboxylate

Given the high instability of gallate and the significant accumulation of 3MGA in the media during ADP1 WT cultivations before it is further *O-*demethylated to gallate, we opted to establish a PDC production pathway directly from 3MGA. Using 3MGA as a product precursor instead of gallate also eliminates the need for the second *O*-demethylation step. To that end, we expressed a gallate dioxygenase, *galA*, from *P. putida* KT2440 in ADP1. GalA is known to convert 3MGA into 4-carboxy-2-hydroxy-6-methoxy-6-oxohexa-2,4-dienoate (CHMOD) which is non-enzymatically transformed into PDC [30]. We cloned *galA* into pKLxR5 backbone under LuxR/P_*luxB*_ promoter, and the expression plasmid was transformed into ADP1 WT, resulting in the strain ASA1005. In addition to the activity of GalA, small amounts of gallate may also be converted into PDC by the native PcaHG. Next, we compared ASA1005 expressing *galA* with and without induction to ADP1 WT in the same conditions. With induction, ASA1005 produced PDC from syringate with a yield of approximately 0.38 mol_PDC_/mol_syringate_ (Fig. 8). Only trace amounts of gallate were detected from the media with induced ASA1005, possibly due to oxidation of gallate into oxalomesaconate by GalA or the abiotic degradation or polymerization of gallate, although no dark coloration indicative of this was observed in the cultures.

Following the confirmation of PDC production by the strain ASA1005, we aimed to investigate the conversion dynamics of 3MGA to PDC by GalA under more controlled conditions. To this end, we performed fed-batch bioreactor cultivations. We hypothesized that maintaining a surplus of syringate in the bioreactor would promote the accumulation of 3MGA, thereby limiting its conversion to gallate. As previously discussed, gallate accumulation leads to the formation of black precipitate and prevents its further conversion. In addition, glucose supplementation supporting the primary metabolism could enhance the demethylation reactions. Given that too high 3MGA concentrations are known to inhibit GalA activity [30], it may be beneficial to have a higher expression level of *galA* in relation to *vanAB*. Thus, 10 µM AHL concentration was chosen for the induction in the subsequent experiments. The initial bioreactor medium was supplemented with 2 mM syringate, 10 mM glucose and 10 µM AHL. The feed contained the same components, except for increased concentrations of syringate (20 mM) and glucose (75 mM), resulting in feeding rates of 0.061–0.067 mmol/h and 0.23–0.25 mmol/h, respectively.

**Fig. 9:**
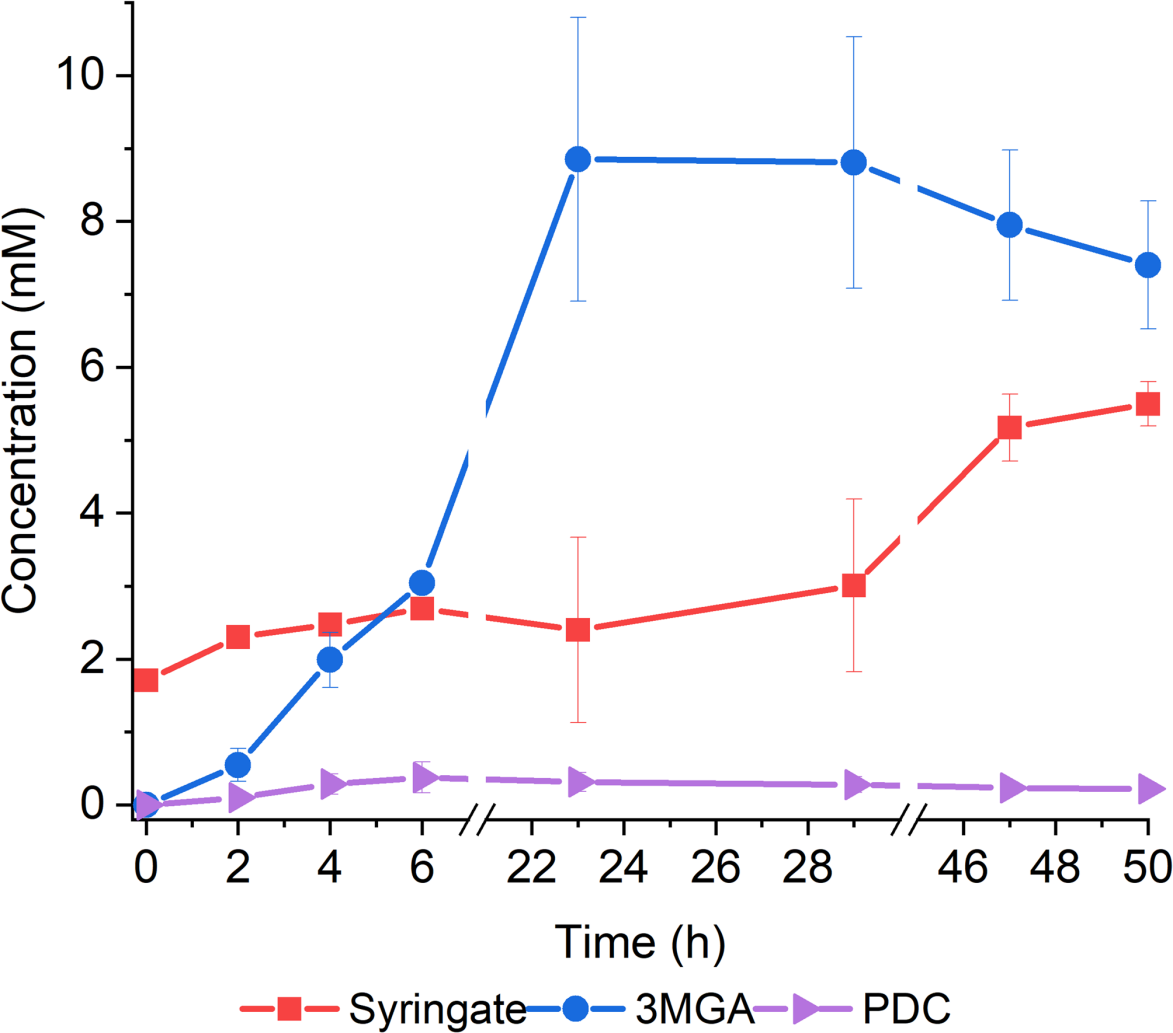
PDC production in bioreactor from syringate with ASA1005. The reactor was maintained at 30 °C, 200 rpm, and at constant aeration. Starting volume of the cultivations was 50 ml of MSM media supplemented with 10 mM glucose, 0.2% casein amino acids, 2 mM syringate, 10 µM AHL, 15 µg/ml gentamicin, and inoculant, resulting in initial OD of approximately 4. Syringate and glucose were fed to the reactors at rates of 0.061–0.067 mmol/h and 0.23–0.25 mmol/h, respectively. Feeding was started at 1 h. Mean values and standard deviations are calculated from two parallel cultivations.3MGA: 3-O-methylgallate, PDC: 2-pyrone-4,6-dicarboxylate

As predicted, continuous syringate feeding effectively prevented the conversion of 3MGA into gallate (Fig. 9, Additional file 1, Figure S11-12). However, potentially due to the significant 3MGA accumulation, the production of PDC remained low, with a yield of approximately 0.031 mol_PDC_/mol_syringate_ after 50 h. PDC concentration increased primarily during the first 6 h after which it remained at a similar level. Dissolved oxygen levels in the cultures indicated that cell growth ceased between 15 and 20 h (Additional file 1, Figure S12).

**Fig. 10:**
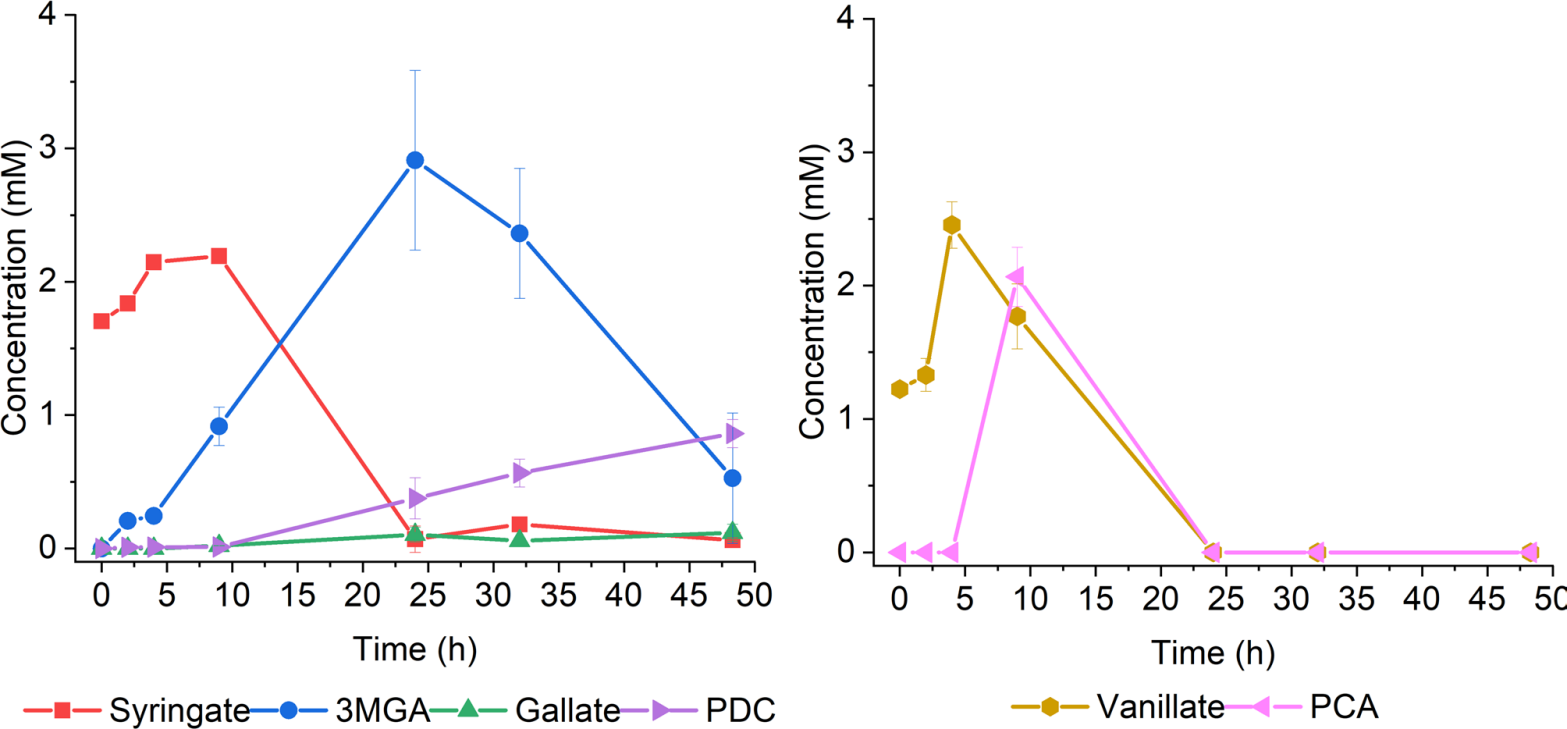
PDC production in bioreactor with vanillate supplementation. ASA1005 was cultivated with co-supplementation of syringate and vanillate. The reactor was maintained at 30 °C, 200 rpm, and at constant aeration. Starting volume of the cultivations was 50 ml of MSM media supplemented with 10 mM glucose, 0.2% casein amino acids, 2 mM syringate, 2 mM vanillate, 10 µM AHL, 15 µg/ml gentamicin, and inoculant, resulting in initial OD of approximately 4. Vanillate, syringate and glucose were fed to the reactors at rates of 0.052–0.055 mmol/h, 0.017–0.018 mmol/h and 0.26–0.27 mmol/h, respectively. Feeding was started after 1 h. At 73 h 0.45 mmol of syringate and vanillate were added to the reactors. Mean values and standard deviations are calculated from two parallel cultivations. 3MGA: 3-O-methylgallate, PDC: 2-pyrone-4,6-dicarboxylate

Based on these results, we hypothesized that co-supplying vanillate alongside syringate might similarly inhibit VanAB-mediated conversion of 3MGA into gallate. Furthermore, reducing syringate levels could mitigate 3MGA accumulation and its associated inhibitory and toxic effects. Accordingly, we performed another bioreactor cultivation (Fig. 10, Additional file 1, Figure S13-14) with initial media supplemented with 2 mM syringate, 2 mM vanillate, 10 mM glucose, and 10 µM AHL. The feed contained 15 mM vanillate, 5 mM syringate, 75 mM glucose, and 10 µM AHL, resulting in feeding rates of 0.052–0.055 mmol/h, 0.017–0.018 mmol/h, and 0.26–0.27 mmol/h, respectively.

This modified setup significantly improved PDC production in the bioreactor, achieving a yield of 0.19 mol_PDC_/mol_syringate_ after 48 h of cultivation. Only trace amounts of gallate were detected, suggesting that some gallate may have been converted into OMA by GalA, as the amount of PDC did not fully account for the consumed 3MGA. Lower 3MGA concentrations appeared to support better cell growth, with activity sustained until 85 h (Additional file 1, Figure S14). Notably, syringate and vanillate were consumed simultaneously in this setup, demonstrating the viability of this strategy suitable for directing syringate metabolism.

## Discussion

ADP1 has shown great promise for the utilization of lignin-related aromatic compounds [40, 42–47]. However, *O-*demethylation of S-lignin derived aromatics by ADP1 has not been previously detected. In this study, we investigated the reactions carried out by VanAB, a promiscuous vanillate *O-*demethylase. Other VanAB homologs can *O-*demethylate many S-lignin derived aromatics, as has been demonstrated in *Pseudomonas* sp. HR199 (syringate) [32], *Streptomyces sp*. NL15-2 K (syringate and 3MGA) [34] in *P. putida* KT2440 (syringate and 3MGA in the presence of an auxiliary carbon source) [30, 33] and most recently in in *Pseudomonas* sp. NGC7 [35]. In this context, more extensive study of VanAB of ADP1 seemed warranted despite the fact that previously activity for syringate or 3MGA was not observed [36].

First, we examined how non-native expression of *vanAB* affects the growth of ADP1 on vanillate, a native carbon source of ADP1 (Fig. 2). As expected, the deletion of *vanAB* (strain ASA1002) prevented growth on vanillate as a sole carbon source. The strains overexpressing *vanAB* had significantly longer lag phases compared to ADP1 WT, and based on OD, the final biomasses were also slightly lower. Of the engineered strains, the uninduced ASA1004 strain performed the best, supporting the previous findings [30] that low expression level of *vanAB* is beneficial in terms of growth. The negative effects of *vanAB* overexpression on the growth can be explained by the increased NAD(P)H consumption and the rapid generation of formaldehyde formed in the *O*-demethylation reaction [29]. In *P. putida* KT2440, providing additional carbon and energy source for the cells significantly improved growth on syringate with strains overexpressing *vanAB*, likely due to increased supply of cofactors [30]. In addition, overexpression of *vanAB per se* can potentially have negative impact on growth, although such effect was not observed in ADP1 while producing VanAB for in vitro studies, when specific substrate for VanAB was not available.

In the ADP1 WT, *vanAB* is repressed by a GntR-type transcriptional regulator VanR, which typically causes a 3–4-hour lag-phase preceding vanillate conversion [66]. Overexpression of *vanAB* in strains ASA1003 and ASA1004 resulted in approximately 10-hour long lag-phase, despite that the expression is not under the regulation of VanR. Thus, the longer lag phase is potentially related to the redox-imbalance and toxicity of formaldehyde, as discussed above.

Next, we explored syringate tolerance of the ADP1 strains. At a concentration of 20 mM, syringate caused an extended lag phase in all strains. In concentrations ranging from 1 to 10 mM, we observed that the overexpression of *vanAB* influences syringate tolerance and growth. For ASA1002 with *vanAB* deletion, the impact of syringate on the growth was more clearly dependent on the concentration. In contrast, for ADP1 WT and uninduced ASA1004, the inhibitory effect of syringate was similar in all concentrations. Notably, when *vanAB* was overexpressed in ASA1004, syringate had a drastic effect on growth even at low concentrations. The growth of the induced ASA1004 was potentially hindered by the faster *O-*demethylation of syringate, similarly to what was observed in vanillate cultivations.

Regardless, all the strains were able to grow in the concentrations tested. Tolerance to even higher concentrations could be further explored and improved in the future, for example by adaptive laboratory evolution, as we have previously demonstrated with ferulate [48]. Growth in up to 120 mM syringate has been reported for *P. putida* KT2440, with overexpression of *vanAB* increasing tolerance [30]. However, in ADP1, gallate is not further metabolized and its accumulation potentially adds to the toxicity of syringate.

To explore the reactions carried out by natively expressed *vanAB* on non-native carbon sources, we set up cultivations of ADP1 WT with 5 mM syringate and 50 mM glucose supplementations. We found that syringate is quickly *O-*demethylated into 3MGA and then into gallate by ADP1, in contrast to previous research where no activity for either substrate was detected [36]. The discrepancy with earlier VanAB characterization in ADP1, as reported by Morawski et al. [36], could be attributed to differences in experimental methods. In their study, whole cell bioconversion of the different VanAB substrates was carried out with ADP1 and recombinant *E. coli* expressing VanAB from ADP1. VanAB activity in ADP1 was measured using washed cell suspensions with the tested VanAB substrates as sole carbon sources. This setup, which lacks the mechanisms to replenish intracellular NAD(P)H reservoirs, may have significantly reduced the overall reaction capacity. In our experiments, we included glucose as an auxiliary carbon source to support both cellular growth and NAD(P)H replenishment. Furthermore, recombinant expression systems may not fully replicate the native conditions, potentially leading to altered or incomplete protein functionality. This challenge is particularly evident in the expression of VanAB enzymes, where solubility issues have been reported for the recombinantly expressed VanAB from *Streptomyces sp.* NL15-2 K and *Pseudomonas sp.* HR199 [32, 34]. These solubility limitations may explain the absence of detectable activity towards syringate or 3MGA in the study by Morawski et al. [36]. Taken together, to accurately assess substrate-specific activities of VanAB, it is essential to examine enzyme variants within their native host organisms under conditions that ensure sufficient NAD(P)H availability. Such an approach would provide a more reliable representation of enzymatic function and substrate specificity.

We detected high amounts of 3MGA in the ADP1 WT culture media prior to its further conversion to gallate. The demonstrated dynamic conversion, in which syringate is *O-*demethylated first is consistent with previous findings that suggest 3MGA is less preferred substrate for VanAB [30, 32]. For comparison, *P. putida* KT2440 which natively *O*-demethylates syringate, was included in the experiments. In *P. putida* KT2440, syringate conversion was much slower than in ADP1 and 3MGA was not *O-*demethylated at all, being in line with the previous study [30]. However, when vanillate was provided instead of glucose for *P. putida* KT2440, a higher syringate conversion rate was achieved, and with continuous vanillate feeding, both syringate and 3MGA were consumed completely [30]. Thus, in *P. putida* KT2440, syringate conversion could be affected by glucose-induced carbon catabolite repression. For ADP1, neither glucose nor gluconate have been observed to repress the utilization of aromatic compounds [46, 50].

As previously mentioned, VanR is a negative transcriptional regulator that represses the expression of *vanAB* in the absence of an inducer, namely vanillate [66, 67]. In our experiments, syringate was *O-*demethylated by VanAB robustly without induction by vanillate in ADP1 WT. Therefore, it appears that syringate can serve as an inducer for *vanAB* expression in ADP1. Amino acid sequence identity of VanR in ADP1 and *P. putida* KT2440 is 48%. Difference in the protein structures or potentially even the different gene organization of the *van* region could affect VanR binding to DNA or VanAB substrates and partially explain the differences in performance between *P. putida* and ADP1.

Next, we investigated syringate conversion by the strains overexpressing *vanAB* along with ASA1002 with *vanAB* deletion as a control. We found that overexpression enables simultaneous conversion of syringate and 3MGA, but for overall efficiency the lower expression level is better. As discussed above, a possible reason for this could be that the *O-*demethylation reaction catalyzed by VanAB produces formaldehyde as a side product, which has been previously suggested to cause a redox imbalance and energy limitation in *P. putida* KT2440 [30].

A conserved route for detoxification of formaldehyde utilizes a glutathione-dependent formaldehyde dehydrogenase and a S-formylglutathione hydrolase [29]. Formaldehyde detoxification during syringate catabolism was recently investigated in *Pseudomonas sp*. NGC7 [35]. It was found that an inducible thiol-independent formaldehyde dehydrogenase FdhA2 and constitutively expressed glutathione-dependent formaldehyde dehydrogenase FrmA and hydrolase FrmC play a significant role. Deletion of both genes inhibited growth on syringate, whereas deleting *frmAC* delayed growth. In addition, *fdhA2* was induced in the presence of syringate. While the specific mechanism for eliminating formaldehyde in ADP1 has not been described, in *A. baumannii*, a close relative of ADP1, glutathione-dependent formaldehyde dehydrogenases *adhC1* and *adhC2* have been characterized [68]. Interestingly, the expression of *adhC1* in *A. baumannii* was found to be repressed in the presence of free inorganic iron [68]. This could potentially affect the balance between *O-*demethylation and formaldehyde detoxification, as VanAB requires iron in the iron-sulfur cluster [2Fe-2 S] and in the active site [26]. Notonier et al. [30] supplemented *P. putida* KT2440 cultures with formate, which is oxidized in cells to generate NADH and thus improved the *O-*demethylation of syringate. For ADP1, we found no benefit from formate supplementation (data not shown). This can be potentially attributed to the difference in VanAB preference for NADPH over NADH, although this has not been experimentally studied in ADP1 [29]. In the future, identifying how formaldehyde is detoxified in ADP1 could help to overcome the inhibitory effects caused by *vanAB* overexpression.

In addition to the in vivo experiments, activity of VanAB was evaluated *in vitro.* We were able to co-purify VanA and VanB with a N-terminal His-(6)-tag only in VanA, suggesting strong interaction between them. By the assay setup we showed that NADH consumption can be used to indirectly measure VanAB activity. We found that syringate is nearly as preferred substrate as vanillate. However, in contrast to the in vivo experiments, no activity for 3MGA was detected. To the best of our knowledge, in vitro activity towards 3MGA has been previously shown only for the VanAB homolog from *P. putida* KT2440 and with clear preference for vanillate and syringate as substrates over 3MGA [30]. The purification and assay conditions here may have further negatively impacted the activity, as Rieske oxygenases are known to be sensitive to oxidation [32, 69]. Addition of sources of iron and sulfur, as well as dithiothreitol as an antioxidant have been shown to be beneficial, and systematic characterization of their effects in ADP1 could further improve the activity [32].

The robust and complete *O-*demethylation of syringate and 3MGA in the in vivo experiments yielded approximately equimolar amounts of gallate in ADP1. Gallate itself is an interesting product, as it can be used for example in the production of antioxidants, pharmaceuticals and antimicrobials [70, 71]. However, gallate is known to oxidize readily, indicated by the change of color to dark brown or black (Supplementary Figure S3, Additional File 1). Gallate can also react with media components and proteins and may form a precipitate that could be harmful for cell growth and prevent its further utilization [7, 63]. To that end, we wanted to explore further ways to utilize the robust and dynamic *O-*demethylation of syringate and 3MGA for production in ADP1.

Directing 3MGA into PDC by the expression of *galA* emerged as an attractive option as it would benefit from the accumulation of 3MGA that occurs when *vanAB* is natively expressed in ADP1. Conversion of 3MGA directly into PDC by GalA would also be beneficial because it circumvents the second *O-*demethylation step, reducing NAD(P)H consumption and production of toxic formaldehyde. Reported rate for this reaction has been low, in part due to substrate inhibition by 3MGA [30], but it had not been tested in ADP1. An alternative route would be via PcaHG found both in ADP1 and *P. putida* KT2440, in which it converts gallate into PDC. Despite that we observed only modest conversion of gallate to PDC in ADP1 by PcaHG, it can potentially be exploited to support PDC production in combination with GalA: any 3MGA that is not directly converted into PDC by GalA could be salvaged by the conversion of gallate into PDC by PcaHG.

By introducing *galA* from *P. putida* KT2440 into ADP1, we successfully demonstrated the production of PDC from syringate in ADP1 with a molar conversion yield of approximately 0.38 mol_PDC_/mol_syringate_. To further investigate the syringate conversion dynamics in more controlled conditions, the subsequent cultivations were carried out in a bioreactor. This setup enabled continuous feeding for testing whether maintaining a consistent syringate concentration in the medium could limit the *O-*demethylation of 3MGA and thereby reduce gallate production. At the same time, glucose was provided as a supporting carbon source to promote cell growth and metabolic activity.

Indeed, we found that maintaining a high concentration of syringate in the media effectively suppressed the conversion of 3MGA to gallate. However, despite this, PDC production remained unexpectedly low, which we attribute to the limited conversion of 3MGA to PDC under the studied conditions. One plausible explanation for this limited conversion is the inhibitory effect of 3MGA on GalA activity. Previous studies have shown that in other extradiol dioxygenases, 3MGA can oxidize the ferrous iron in the enzyme’s active site, leading to inactivation of the enzyme [72]. In addition, cell growth was observed to stagnate between 15 and 20 h (Additional file 1, Figure S12), which may be due to the cytotoxic effects of elevated 3MGA levels. This accumulation could further contribute to the inhibition of GalA activity, compounding the low PDC yields.

To address this, we performed bioreactor cultivations in which vanillate and syringate were co-fed to the reactor. This strategy aimed to balance the conversion of syringate to 3MGA while minimizing its further conversion to gallate, without relying on excessive syringate supplementation. As anticipated, the *O*-demethylation of vanillate and syringate occurred simultaneously, thereby resulting in more stable syringate conversion rate and reduced accumulation of 3MGA. Although the overall PDC yield in the bioreactor (250 ml, 0.19 mol_PDC_/mol_syringate_) was found to be lower than in the small-scale batch experiment (10 ml, 0.38 mol_PDC_/mol_syringate_) within a ~ 50 h timeframe, the cofeeding approach demonstrated the potential to produce PDC in larger scale cultivation in a fed-batch manner. Moreover, cofeeding vanillate and syringate emerged as a viable approach to both limit the undesired conversion of 3MGA to gallate and maintain moderate, sustained levels of 3MGA as a substrate for GalA. Furthermore, the simultaneous conversion of vanillate and syringate by VanAB highlights its robust catalytic activity, which is particularly advantageous in bioprocesses involving complex mixtures of lignin-derived aromatic compounds. This dual substrate utilization not only enhances metabolic flexibility but also supports more efficient valorization of heterogeneous lignin streams.

In future studies, several approaches could be applied to further improve PDC production in ADP1. For example, in this study, we used a *galA* gene directly from the genome of a GC-rich strain *P. putida* KT2440, while a synthetic gene codon optimized specifically for ADP1 could function more optimally. In addition, protein engineering of the substrate specificity of GalA towards 3MGA could be a potential strategy for improving the conversion. Furthermore, employing alternative enzymes such as LigAB and DesZ from *Sphingobium* spp. SYK-6 which can also carry out the ring-opening reaction [73], should be considered.

Beyond syringate upgrading, a key direction for future research involves the simultaneous conversion and valorization of aromatic mixtures that more accurately represent the complexity of lignin-derived biomass. *A. baylyi* ADP1 naturally metabolizes H- and G-type lignin-derived compounds such as *p*-coumarate and ferulate, and has previously been engineered to utilize guaiacol [74]. To fully exploit the metabolic potential of this strain, future efforts will focus on developing integrated metabolic engineering strategies to funnel diverse aromatic substrates from all lignin types into a unified product stream. This could be achieved by introducing heterologous pathways for the utilization of key intermediates in syringate catabolism, such as gallate, thereby enabling the efficient channeling of carbon from mixed aromatic sources into common intermediates or central metabolites. These intermediates could then be further upgraded into value-added products, advancing the feasibility of lignin valorization in biotechnological applications.

## Conclusions

In this study, we established the conversion and upgrading of S-lignin related syringate in *Acinetobacter baylyi* ADP1. We characterized the activity of a promiscuous vanillate *O-*demethylase VanAB in ADP1, demonstrating its efficient *O-*demethylation of two previously unrecognized substrates, syringate and 3MGA. Overexpression of *vanAB* altered the conversion dynamics, enabling simultaneous conversion of 3MGA and syringate, unlike in the ADP1 WT, where syringate is *O-*demethylated first. Excessive expression levels negatively impacted cell growth, likely due to the accumulation of toxic intermediates and the high energy demand of *O*-demethylation, as previously reported. Finally, we exploited the accumulation of 3MGA and introduced gallate dioxygenase GalA from *P. putida* KT2440 to produce PDC, a bioprivileged plastic precursor. By cofeeding vanillate and syringate, we developed a strategy to modulate the balance between syringate and 3MGA *O*-demethylation. This regulation proved beneficial in minimizing the formation of undesired byproducts such as gallate and mitigating the inhibitory effects of 3MGA on both cell growth and GalA activity, thereby enhancing overall process efficiency. Our findings expands the aromatic substrate range of ADP1 to include S-lignin derived aromatics for production and further promotes the use of this host for biological upgrading of lignin-related compounds.

## Supplementary Information

Below is the link to the electronic supplementary material.

Additional file 1. Primers, supplementary results and figures.

## Data Availability

The data that support the findings of this study are included within the articleor the additional files. The corresponding author is willing to provide the raw data related to this manuscript upon reasonable request.

## Abbreviations

ADP1: *Acinetobacter baylyi* ADP1
3MGA: 3-O-methylgallate
AHL: N-(3-oxohexanoyl) homoserine lactone
DTT: Dithiothreitol
GA: Gallate
LA: lysogeny agar
LB: lysogeny broth
MSM: Mineral salts medium
NAD(P)H: Nicotinamide adenine dinucleotide (phosphate)
NMR: ^1^H nuclear magnetic resonance
OD: Optical density
PCA: Protocatechuate
PDC: 2-pyrone-4,6-dicarboxylate
P450: Cytochrome P450
PDA: Photodiode array detector
RO: Rieske oxygenase
SA: Syringate
SDS-PAGE: Sodium Dodecyl Sulfate Polyacrylamide Gel Electroforesis
THF: Tetrahydrofolate
USER: Uracil-Specific Excision Reagent
VA: Vanillate
