# Supplementary material for: Conversion and upgrading of syringate by Acinetobacter baylyi ADP1
Source: Microb Cell Fact. 2025 Sep 29;24:209. doi: 10.1186/s12934-025-02839-1 (PMC12481942; doi:10.1186/s12934-025-02839-1)
Supplement: Supplementary file 1 — Additional file 1. Primers, supplementary results and figures. [file 12934_2025_2839_MOESM1_ESM.docx]

# **Supplemental information for**

# **Conversion and upgrading of syringate by *Acinetobacter baylyi* ADP1**

Heidi Tuomela^1^, Johanna Koivisto^1^_,_ Elena Efimova^1^, Suvi Santala^1*^

^1^Faculty of Engineering and Natural Sciences, Hervanta Campus, Tampere University, Korkeakoulunkatu 8, 33720 Tampere, Finland

**Supplementary tables**

Table S1. VanA from ADP1 (ACIAD0980) and P. putida KT2440 (PP_3736) local alignment with Smith-Waterman, BLOSUM75. Identity: 260/358 (72.63 %), similarity: 295/358 (82.40 %), gaps: 3/358 (0.84 %).


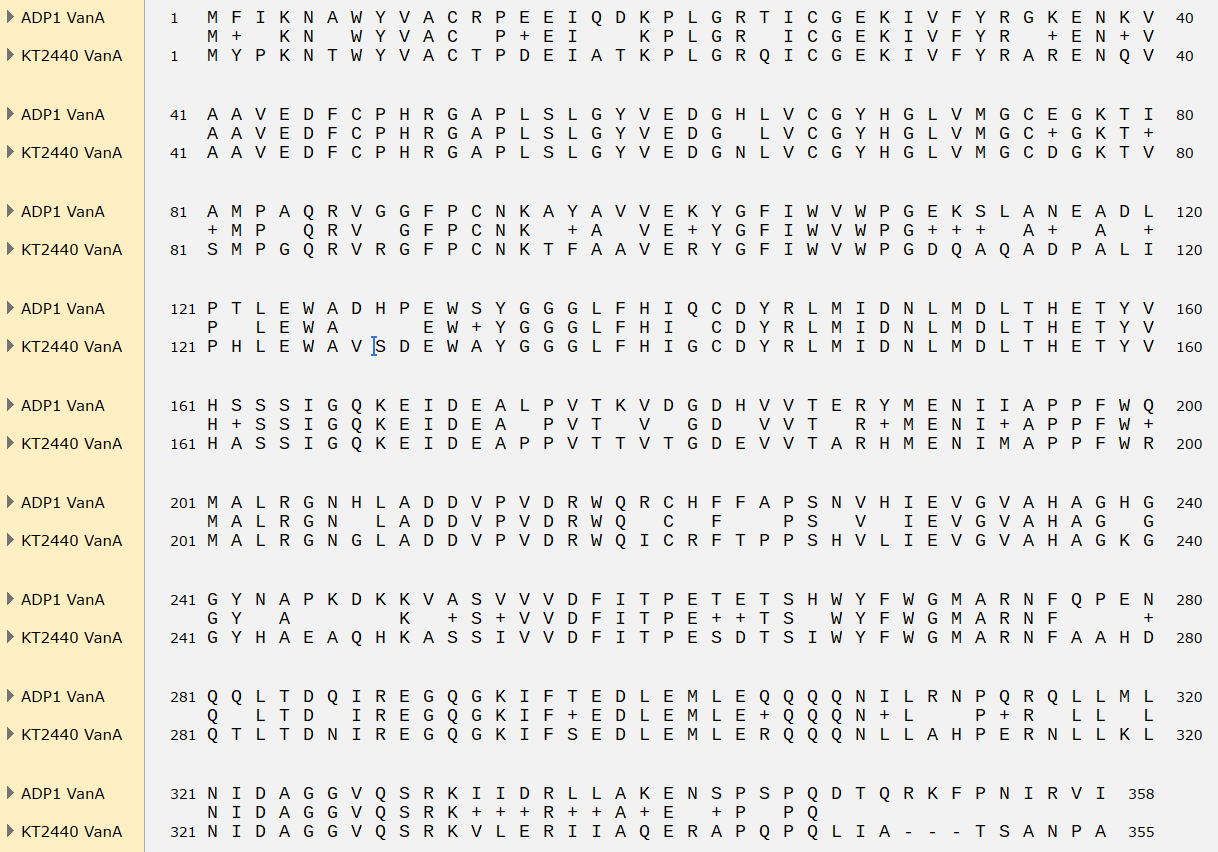


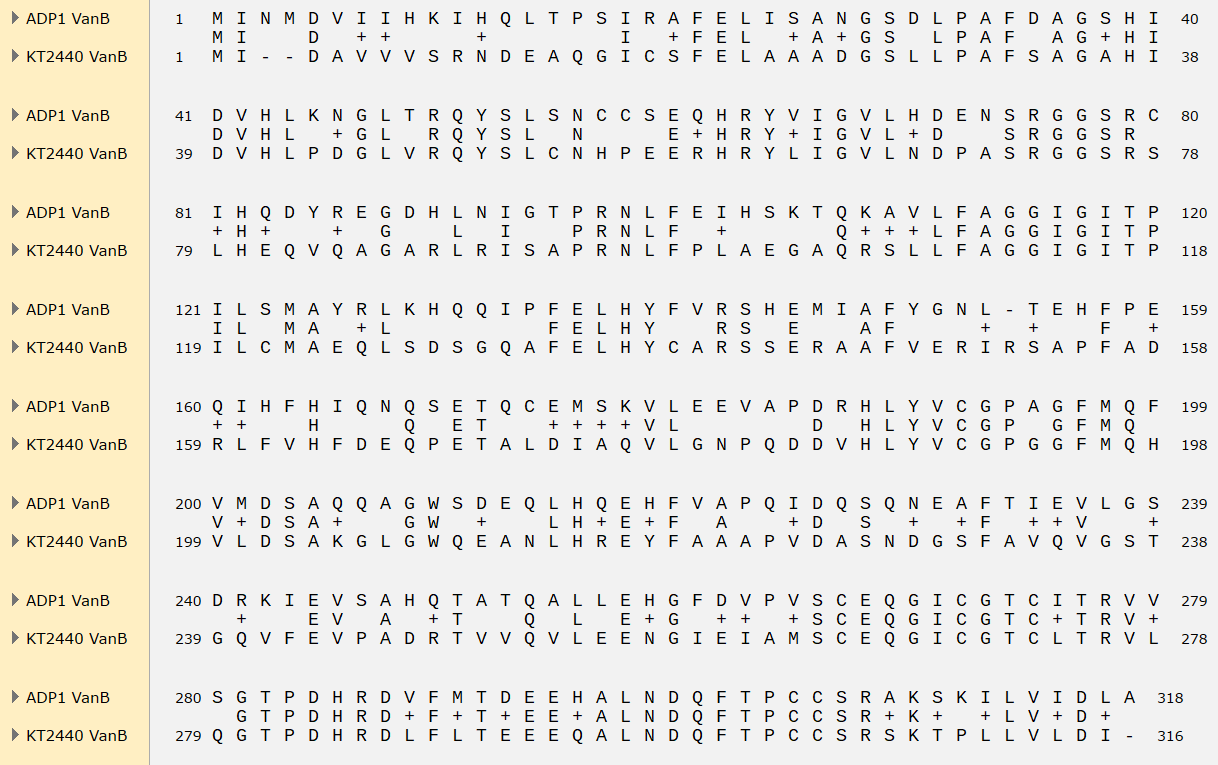


Table S2. VanB from ADP1 (ACIAD0979) and P. putida KT2440 (PP_3737) local alignment with Smith-Waterman, BLOSUM50. Identity: 162/319 (50.78 %), similarity: 218/319 (68.34 %), gaps: 4/319 (1.25 %).

Table S3. VanA from ADP1 (ACIAD0979) and Pseudomonas sp. HR199 (CAA72287) local alignment with Smith-Waterman, BLOSUM70. Identity: 254/358 (70.95 %), similarity: 293/358 (81.84 %), gaps: 4/358 (1.12 %).

**
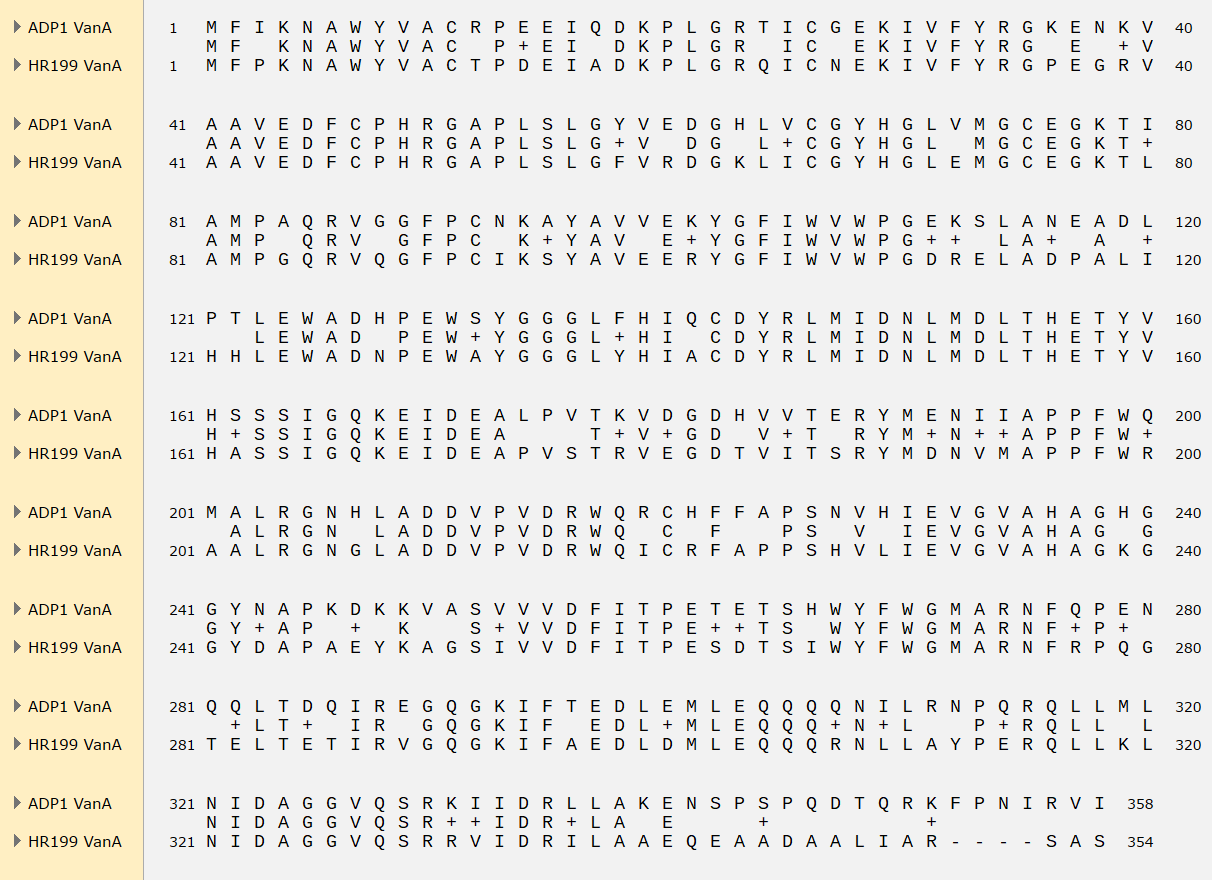
**

Table S4. Primers used in the study.

| Primer | Sequence 5’ 🡪 3 ‘ | Description |
| --- | --- | --- |
| VanAB_F_BB | AATATCTAGAGAAAGAGGAGAAATACTAGATGCAT  CATCATCATCATCACGGTTCTGGTTTTATTAAAAA  TGCCTGGTATGTCGCTTG | *vanAB* insert for  pBAV1Cd-chn, forward, contains sites for BioBrick cloning |
| VanAB_R_BB | AATACTGCAGCGGCCGCTACTAGTATTATTAAGCA  AGATCAATGACTAAAATTTTAGACTTC | *vanAB* insert for pBAV1Cd- chn, reverse, contains sites for BioBrick cloning |
| SS-21-01-VFU | AATAATACUAGTAGCGGCCGCTG | pBAV1C-T5-*vanAB* vector  forward |
| SS-21-02-VRU | ACATCTAGUATTTCTCCTCTTTCTCTAGTAATTGTT  ATCC | pBAV1C-T5-*vanAB* vector  reverse |
| SS-21-03-IFU | ACTAGATGUTTATTAAAAATGCCTGGTATGTC | pBAV1C-T5-v*anAB* insert  forward |
| SS-21-04-IRU | AGTATTATUAAGCAAGATCAATGACTAAAATTTTAG | pBAV1C-T5-v*anAB* insert  reverse |
| VanB R2 | AGGCAAATACCCAGATATTACG | Δ*vanAB* cassette 3’ flanking reverse |
| VanA F2 | CTGTAAATGCCATGACCATC | Δ*vanAB* cassette 5’ flanking forward |
| Tdk_kanF | CCCAGCCTCCAATTCAAATCATAAAAAATTTATTTG | Δ*vanAB* cassette *tdk/kan^r^* forward |
| Tdk_kanR | CCAGCTCCGCATGCTTAGAAAAAC | Δ*vanAB* cassette *tdk/kan^r^* reverse |
| rescue cassette  VanB forward | TCAATTGGCGGCCGCCCTAGGTAAGTTCAGTTTT  TCTCCTATAC | rescue cassette for markerless Δ*vanAB*  forward |
| rescue cassette  VanA reverse | ACCTAGGGCGGCCGCCAATTGAGTGTGACGAC  TCCTTATCAC | rescue cassette for markerless Δ*vanAB*  reverse |
| Ver_VanAB_F | GAAGCCCGATTTGTGCACCTG | Δ*vanAB* verification forward |
| Ver_VanAB_R | AGCGCGTTATAGCTTGGCAT | Δ*vanAB* verification reverse |
| GalA_F | CACTAGCACTATCAGCGTTATCAGTTGGGCGCTTTGCCAGC | To amplify *galA* from *P. putida* KT2440 genome |
| GalA_rev | GCAGATTAAAGAGGAGAAATACTAGATGGCTCGTATCATTGGTGGCCTG | To amplify *galA* from *P. putida* KT2440 genome |
| pKLxR5_fwd | TAACGCTGATAGTGCTAGTGTAGATCGCGCTTTC | To amplify pKLxR5 backbone |
| prR | CATCTAGTATTTCTCCTCTTTAATCTGCGCTCTTCCCAGTTC | To amplify pKLxR5 backbone |

Primer prR from (Schuster and Reisch, 2021)

**
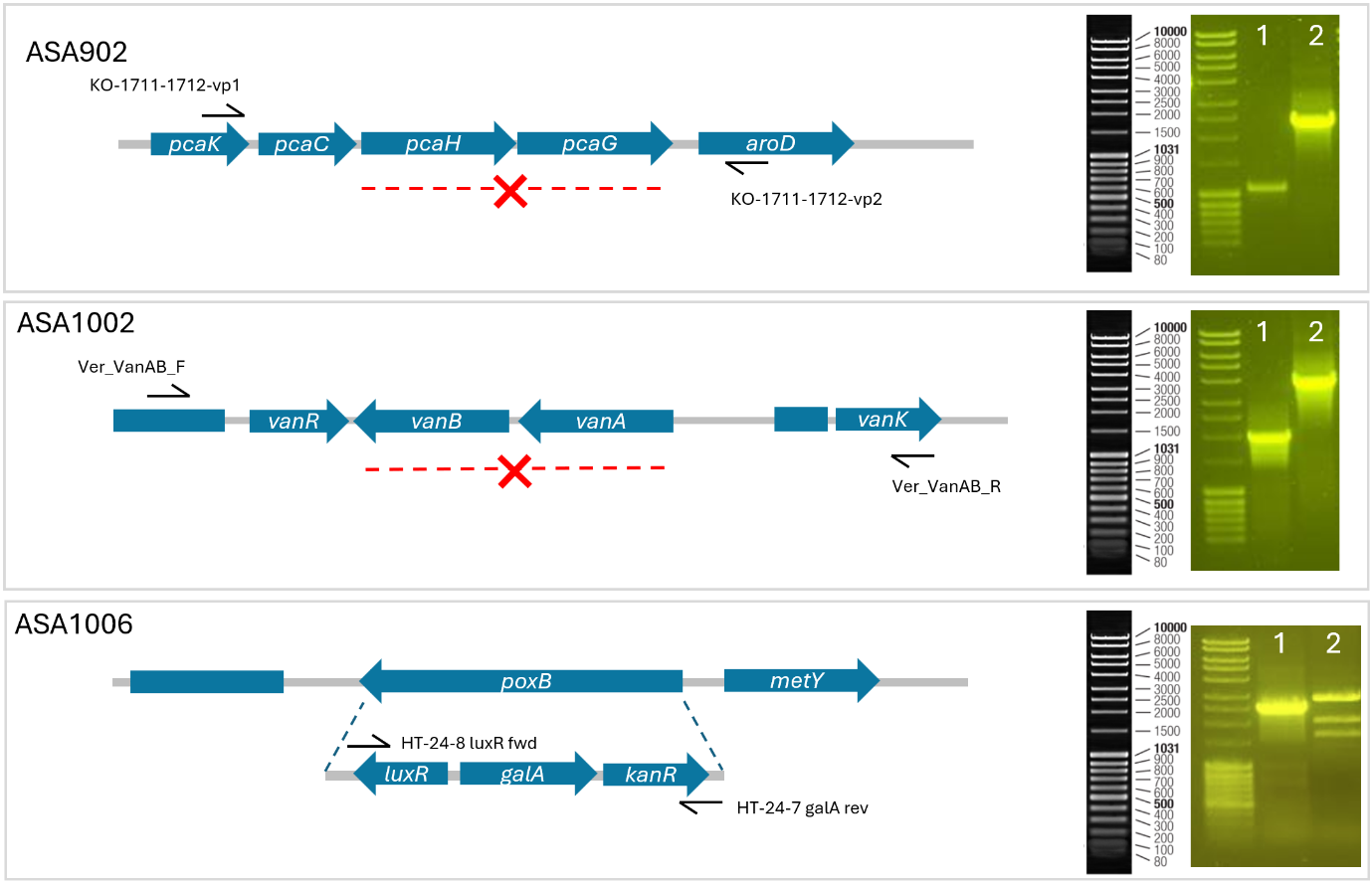
Supplementary figures**

Figure S1. PCR verification of the genomic alterations in ASA1002, lane 1: ASA1002, correct size 2046 bp, lane 2: ADP1 WT as control, correct size 4060 bp


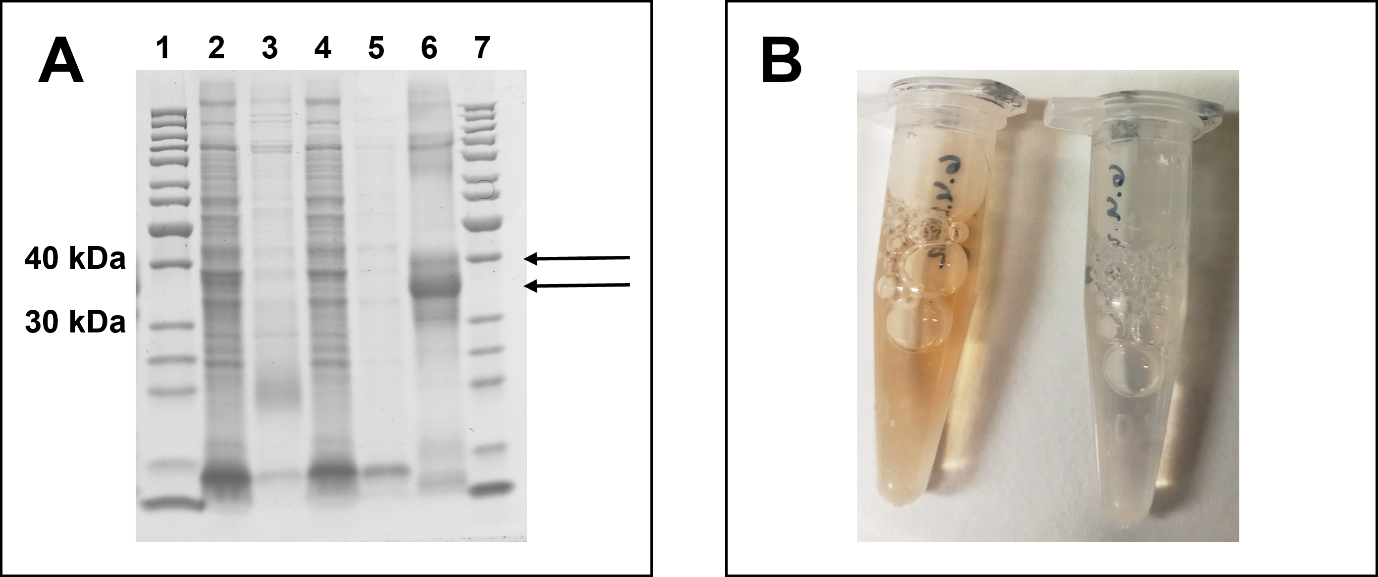
*Figure S2. Typical VanAB purification****.*** *(A) SDS-PAGE gel. Lanes 1 and 7: PageRuler Unstained Protein Ladder (Thermo Fisher Scientific). Lane 2: cleared lysate Lane 3: insoluble fraction Lane 4: column flow-through Lane 5: column wash Lane 6: purified VanAB. VanA ~41 kDa, VanB ~36 kDa. The gel was stained with PageBlue Protein Staining Solution (ThermoFisher Scientific). The image has been modified to grayscale and adjusted for brightness and contrast. (B) Purified VanAB fractions, showing the characteristic red-brown colour of Rieske-type oxygenase****.*** *Left: eluted fraction two, right: eluted fraction three.*


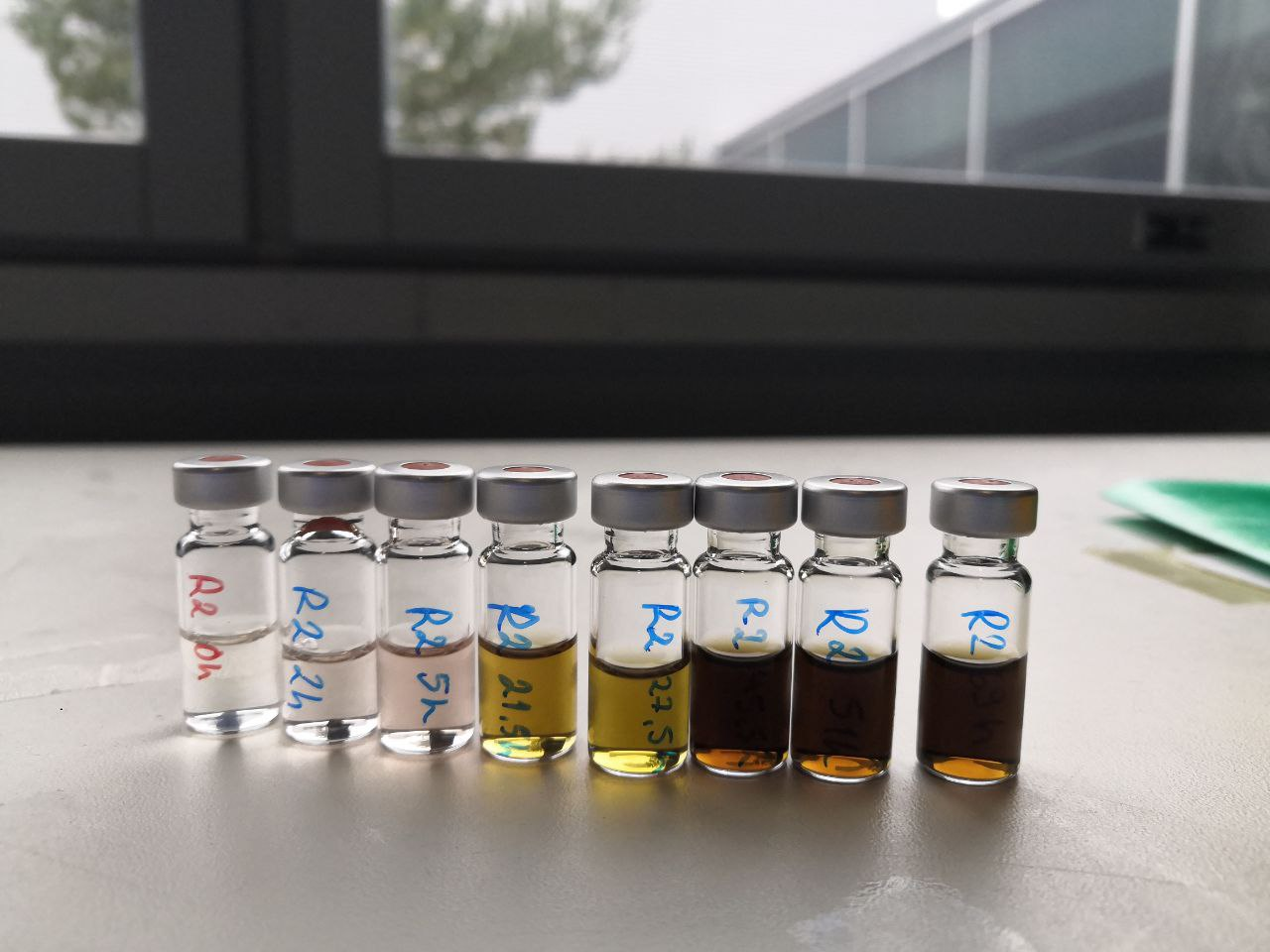

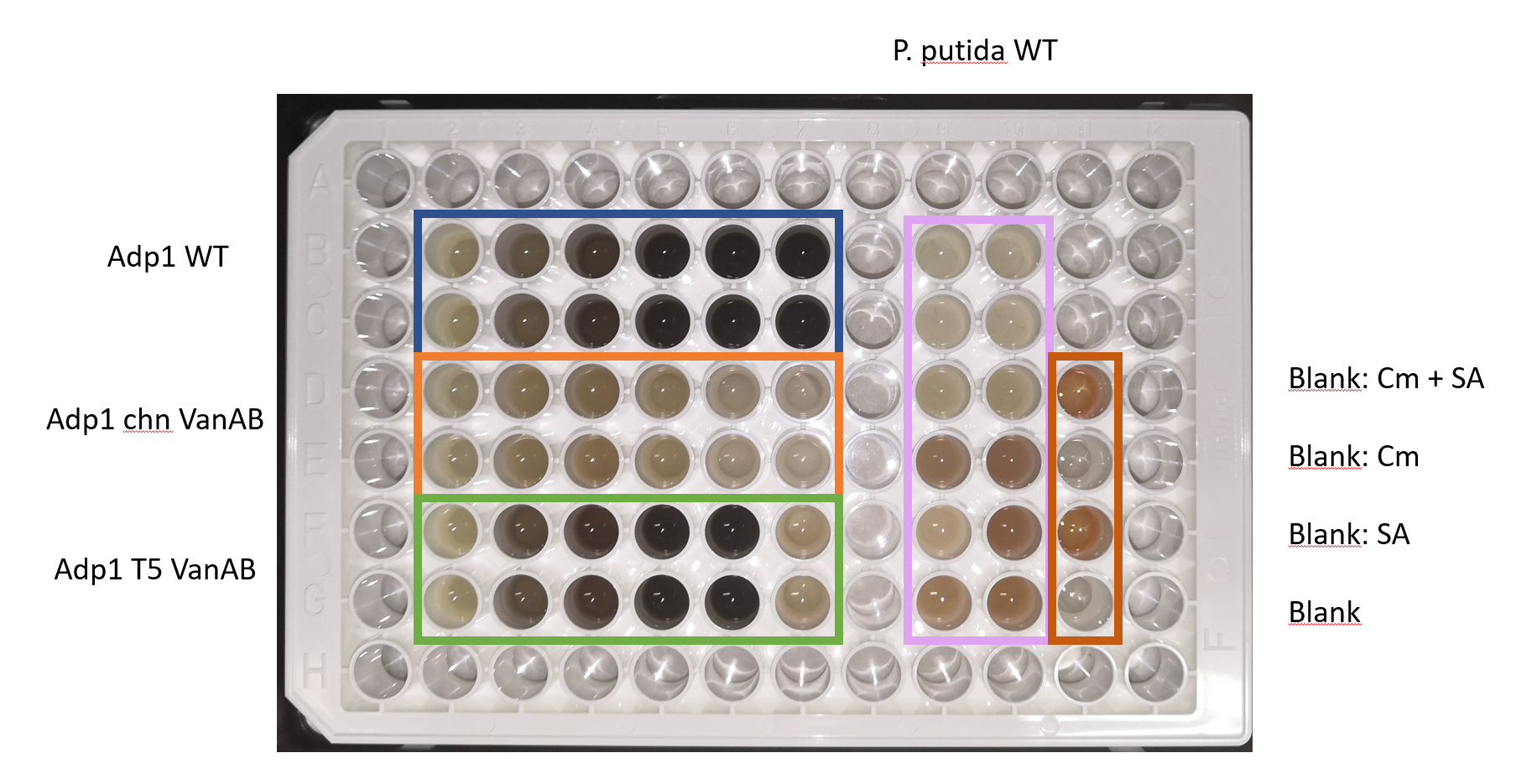


1

2

**E**

**D**

**C**

**B**

**A**

Figure S3. Photos taken from well-plate cultivations, related to the investigation of syringate tolerance (Figure 3) and filtered samples from a bioreactor cultivation. The well-plate (1) depicts an increase in the intensity of the color corresponding to increase in syringate concentration. The bioreactor samples (2) are in chronological order and show the increase in the intensity of dark color, which corresponds with the accumulation of gallic acid. A: ADP1 WT, B: ASA1004, uninduced, C: ASA1003, D: P. putida KT2440. E: media controls.


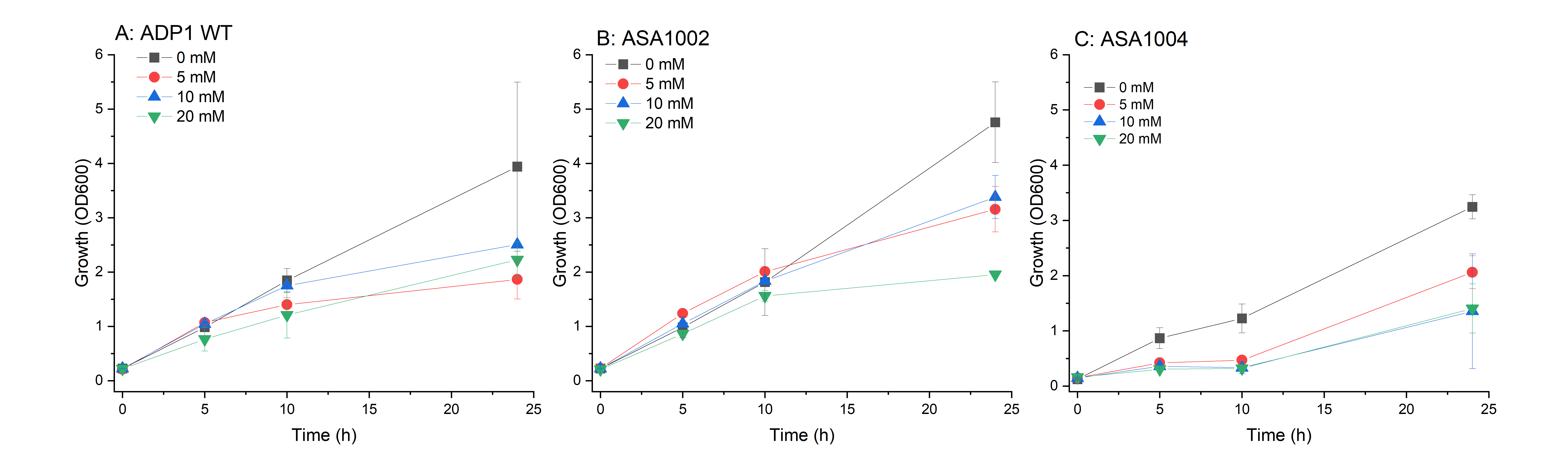


Figure S4. Syringate tolerance. A: ADP1 WT, B: ASA1002, C: ASA1004 induced with 5 µM cyclohexanone. For studying syringate tolerance, strains ADP1 WT, ASA1002 and ASA1004 were precultured overnight in 5 ml volume in MSM media with 0.2% casein amino acids, 20 mM glucose, and appropriate antibiotics. The strains were then cultivated in 14 ml culture tubes in the same media as the precultures supplemented with 0, 5, 10, and 20 mM syringate and 50 mM glucose. Culture conditions were 30 °C and 300 rpm. To measure growth, 1 ml of cells were collected from the cultures and centrifuged at 14 000 rcf for 2 minutes. The supernatant was discarded, and the cells were then washed with PBS buffer before measuring the optical density at 600 nm (OD600). The mean values and error bars of two replicates are shown.


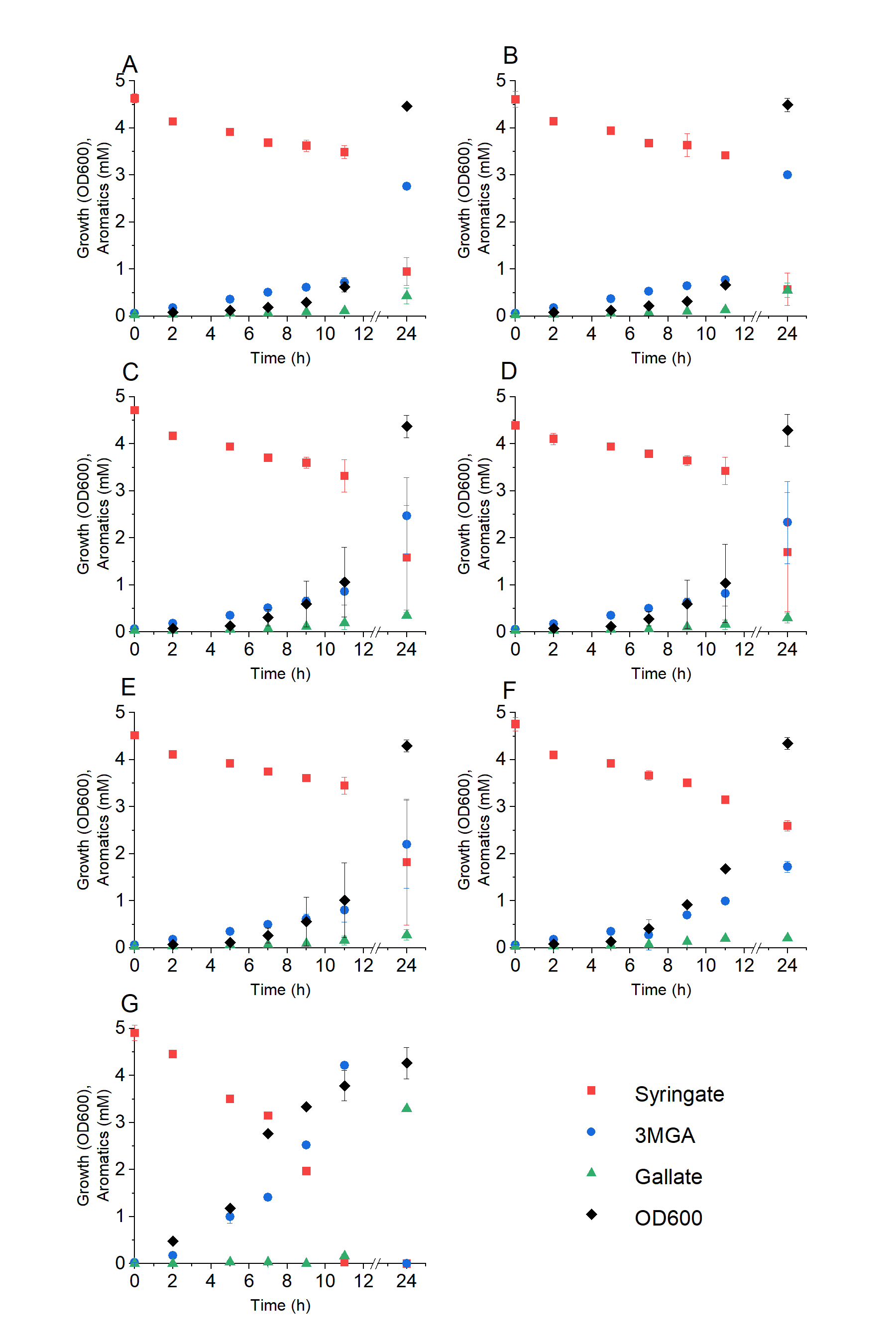


Figure S5. Growth and syringate O-demethylation in ADP1 WT and ASA1004 with different cyclohexanone (cyc) concentrations for inducing vanAB expression. A: ASA1004, 0 µM cyc; B: ASA1004, 0.1 µM cyc, C: ASA1004, 0.5 µM cyc, D: ASA1004, 1 µM cyc, E: ASA1004, 2.5 µM cyc, F: ASA1004, 5 µM cyc, G: ADP1 WT. The strains were cultivated in 50 ml bioreactor tubes in 15 ml mineral salts medium supplemented with 5 mM syringate, 50 mM glucose, 0.2 % casein amino acids, and 25 µg/ml chloramphenicol for ASA1004. Cyclohexanone was added in specified concentrations ranging from 0-5 µM in the beginning of the cultures. Samples were collected at the indicated timepoints to monitor growth (OD600) and metabolite concentrations in the media (analysed with HPLC). The mean values and error bars representing the standard deviations from two parallel cultures are shown. Abbreviations: 3MGA: 3-O-methylgallate, cyc: cyclohexanone.


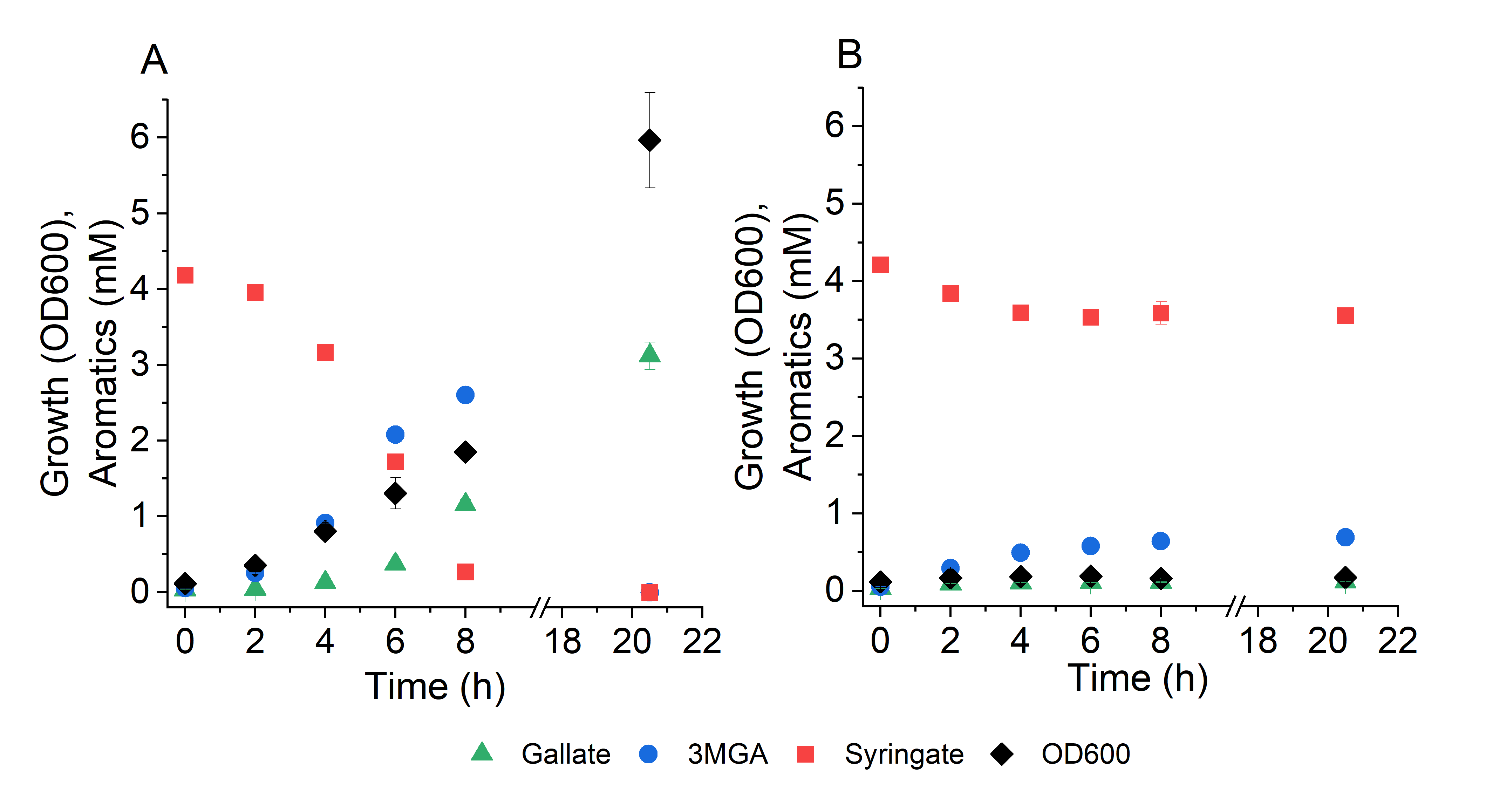


Figure S6. Growth and syringate O-demethylation in ASA1004 with and without cyclohexanone induction. A: ASA1004, B: ASA1004 induced with 100 µM cyclohexanone The strains were cultivated 50 ml bioreactor tubes in 15 ml mineral salts medium supplemented with 5 mM syringate, 50 mM glucose, 0.2 % casein amino acids, and 25 µg/ml chloramphenicol. For the induced culture, 100 µM cyclohexanone was added. Samples were collected at the indicated timepoints to monitor growth (OD600) and metabolite concentrations in the media (analysed with HPLC). The mean values and error bars representing the standard deviations from two parallel cultures are shown. Abbreviations: 3MGA: 3-O-methylgallate.


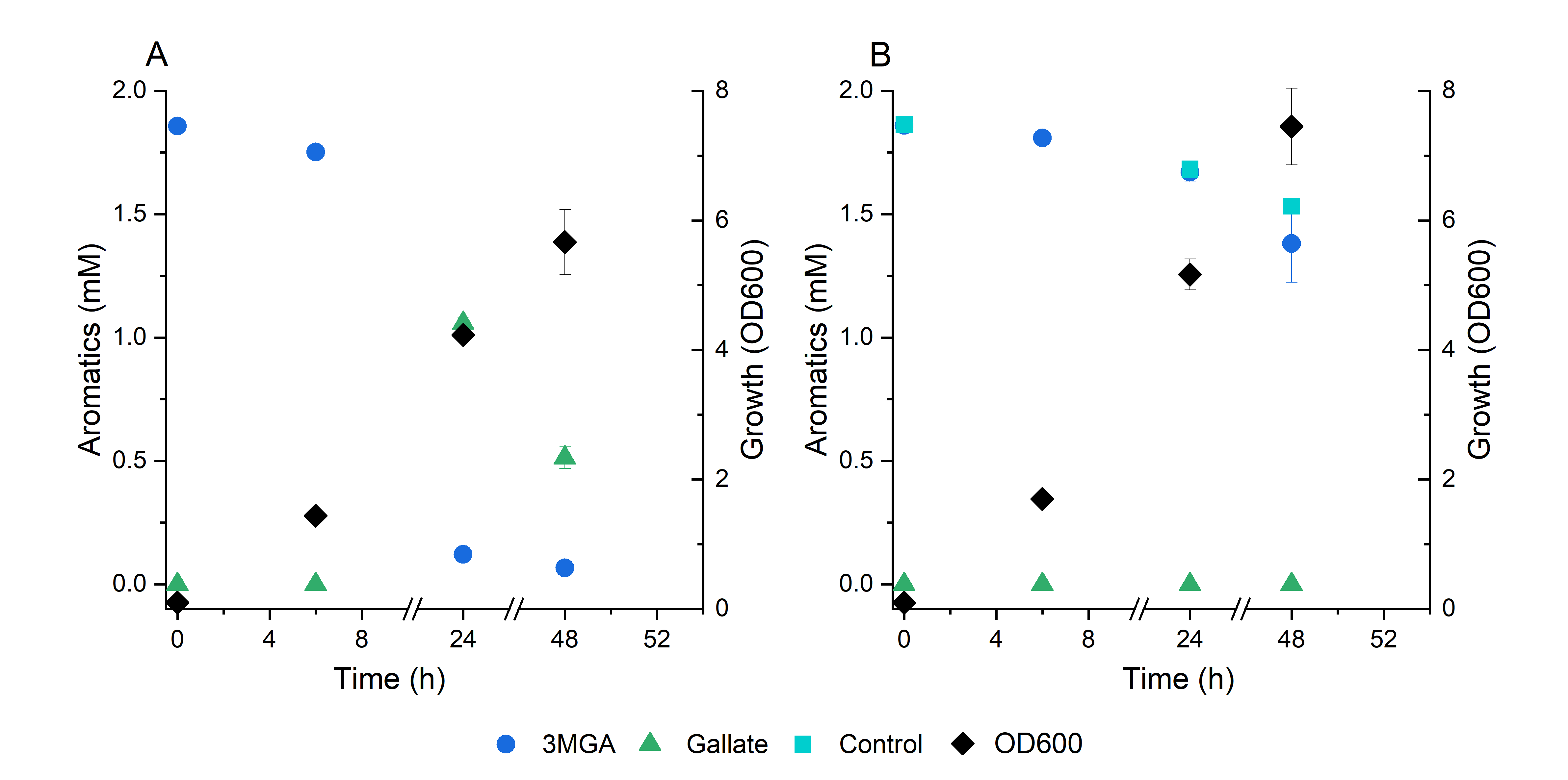


Figure S7. O-demethylation of 3MGA by ADP1 WT and ASA1002. A: ADP1 WT, B: ASA1002 and 3MGA concentration in media control. The strains were cultivated in 50 ml bioreactor tubes in 15 ml MSM supplemented with 2 mM 3MGA, 50 mM glucose and 0.2% casein amino acids. The mean values and error bars representing the standard deviations from three parallel cultures are shown. Abbreviations: 3MGA: 3-O-methylgallate.


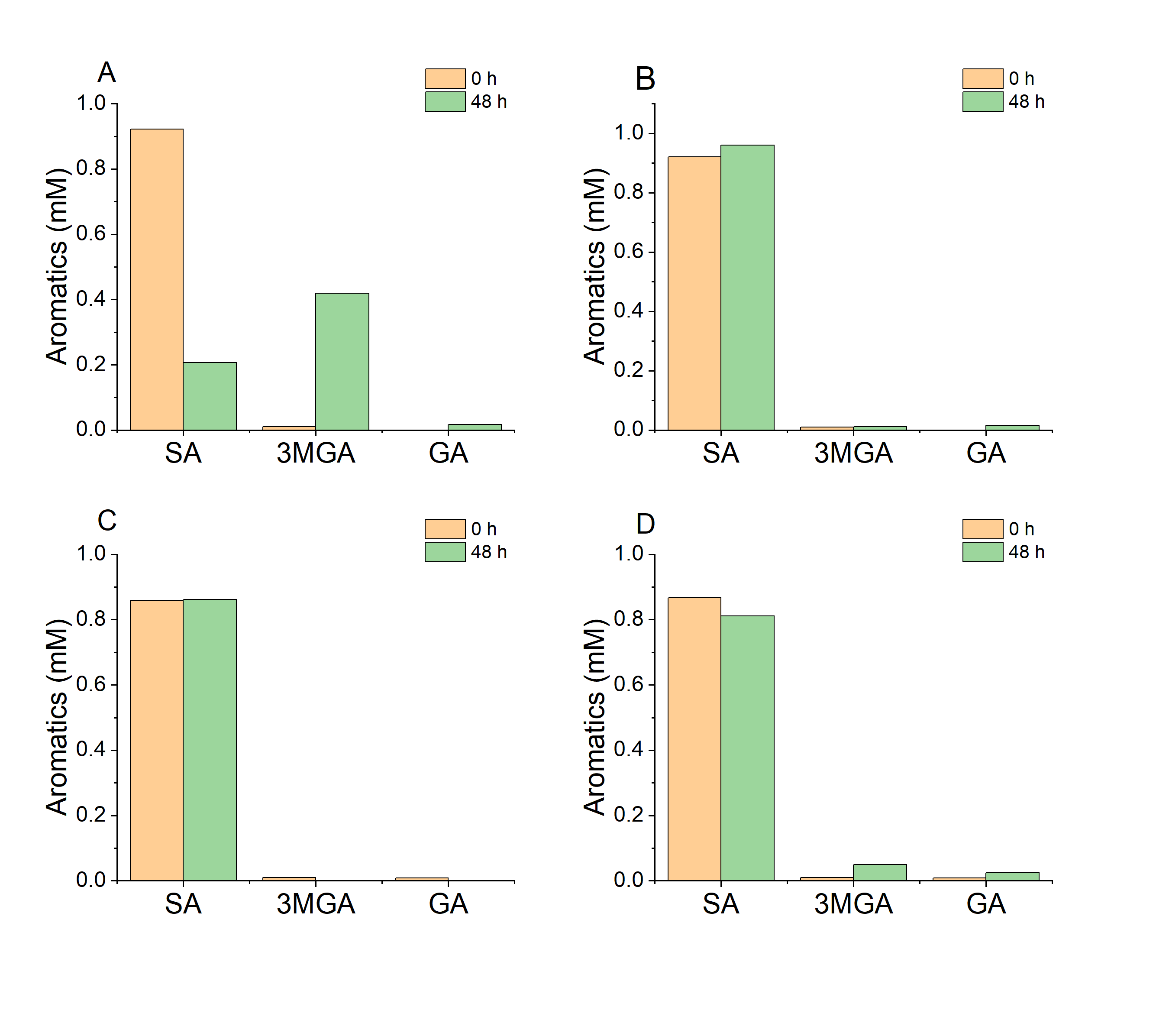


Figure S8. Cultivation of the strains with different constructs on syringate as the sole carbon source, A: ADP1 WT, B: ASA1002, C: ASA1004 induced, D: ASA1004 uninduced. Abbreviations: SA: syringate; 3MGA: 3-O-methylgallate; GA: gallate. The strains were precultivated in MSM media supplemented with 0.2% casein amino acids. Cultivation media consisted of MSM supplemented with 1 mM syringate. In addition, for ASA1004, 25 µg/ml chloramphenicol and 5 µM cyclohexanone were added in the media for selection and induction, respectively. The mean values representing two parallel cultures are shown.

**Gallate utilization by ADP1 WT and ASA1005**

To further investigate the abiotic degradation and to detect possible catabolism of gallate in ADP1, an experiment using gallate as a substrate was carried out as follows: 5 ml precultures inoculated from single colonies on plates were prepared in MSM media supplemented with 20 mM glucose, 0.2 % casein amino acids, and appropriate antibiotics. After overnight incubation at 30 °C, 300 rpm, the cells were inoculated in culture medium to achieve initial optical densities (600 nm) of approximately 0.2. Culture media with MSM and 0.2 % casein amino acids and either 50 mM glucose; 50 mM glucose and 5 mM gallate;5 mM gallate; or control with no additional carbon source were used. Cultures were prepared in triplicates in 15 ml volume and incubated at 30 °C, 300 rpm. Samples from the cultures for OD measurement, HPLC analyses, and gallate stock were prepared as described earlier in the materials and methods section of the manuscript.


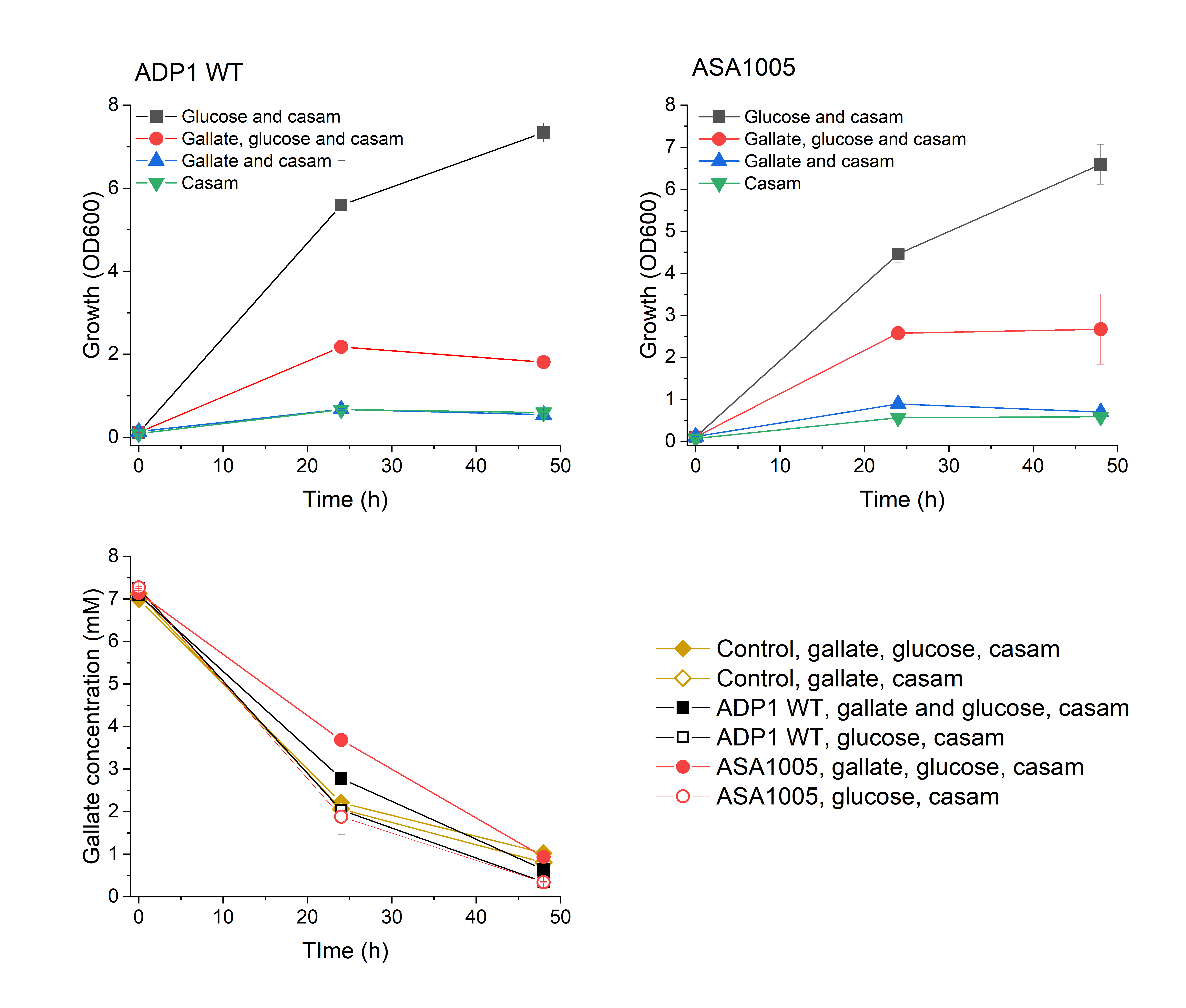


Figure S9. ADP1 WT and ASA1005 cultivations with glucose and casam, gallate, glucose, and casam; gallate and casam; and casam as carbon sources. The graphs on the top row depict the growth of the strains in different media. The third graph shows the decrease in gallate concentrations in cultures with the strains and in the controls without inoculant. The cells were grown in MSM media with appropriate antibiotics, in 15 ml volume, at 30 °C, 300 rpm. The mean values and error bars representing three parallel cultures are shown. Abbreviations, casam: casein amino acids.


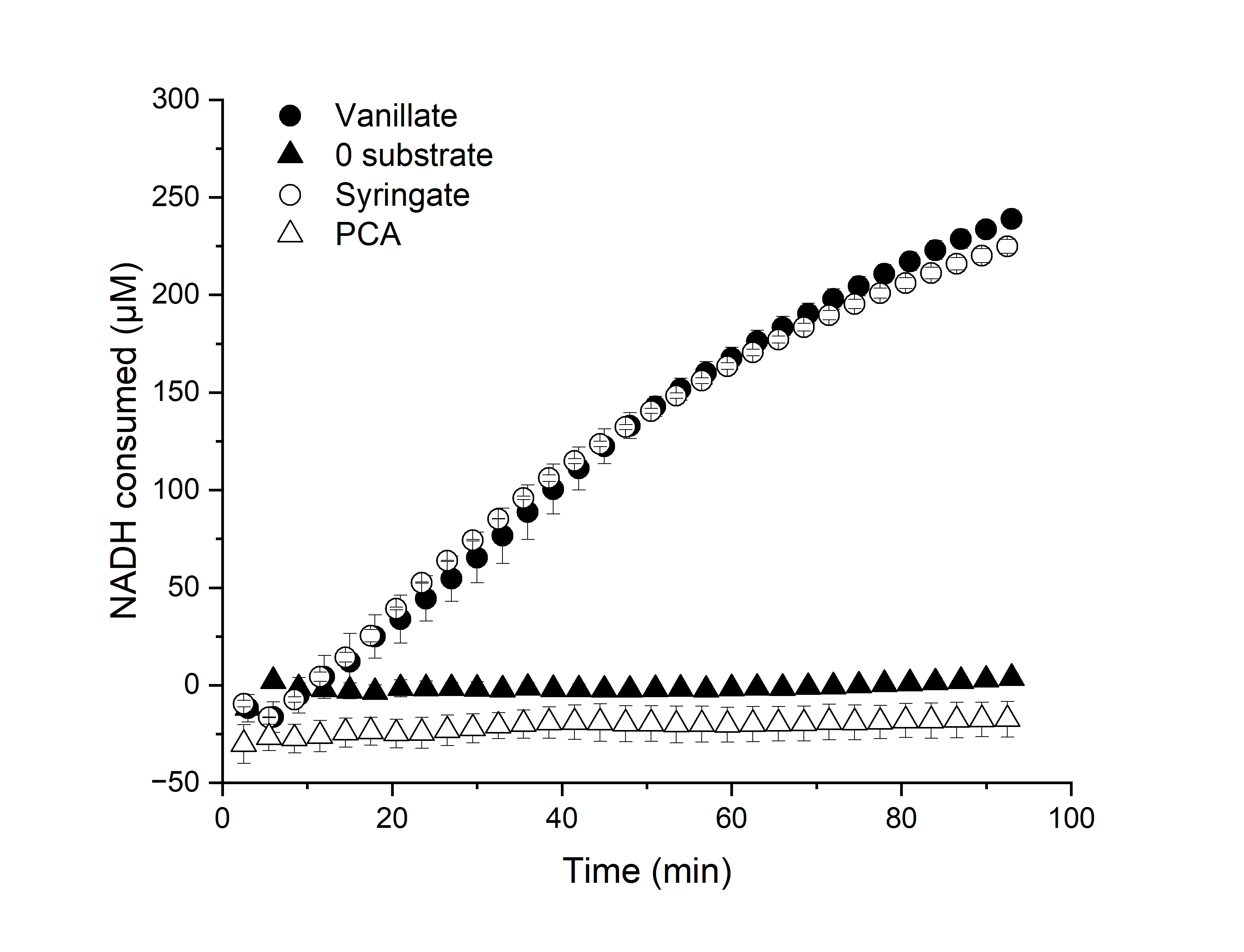


Figure S10. Progress curves of VanAB initiated reactions showing consumption of NADH over time. Vanillate and syringate concentrations in reactions 400 μM. Protocatechuate, PCA, (400 μM) and 0 substrate reactions were conducted as controls, showing only small amount of NADH consumption over time compared to reactions with vanillate or syringate. Reactions were performed in 20 mM Tris-HCl, 100 mM NaCl, 5 mM DTT, pH 8, at 25°C. All reactions contained initial 400 μM NADH. The data is presented as means of duplicate reactions. Error bars represent standard deviation.


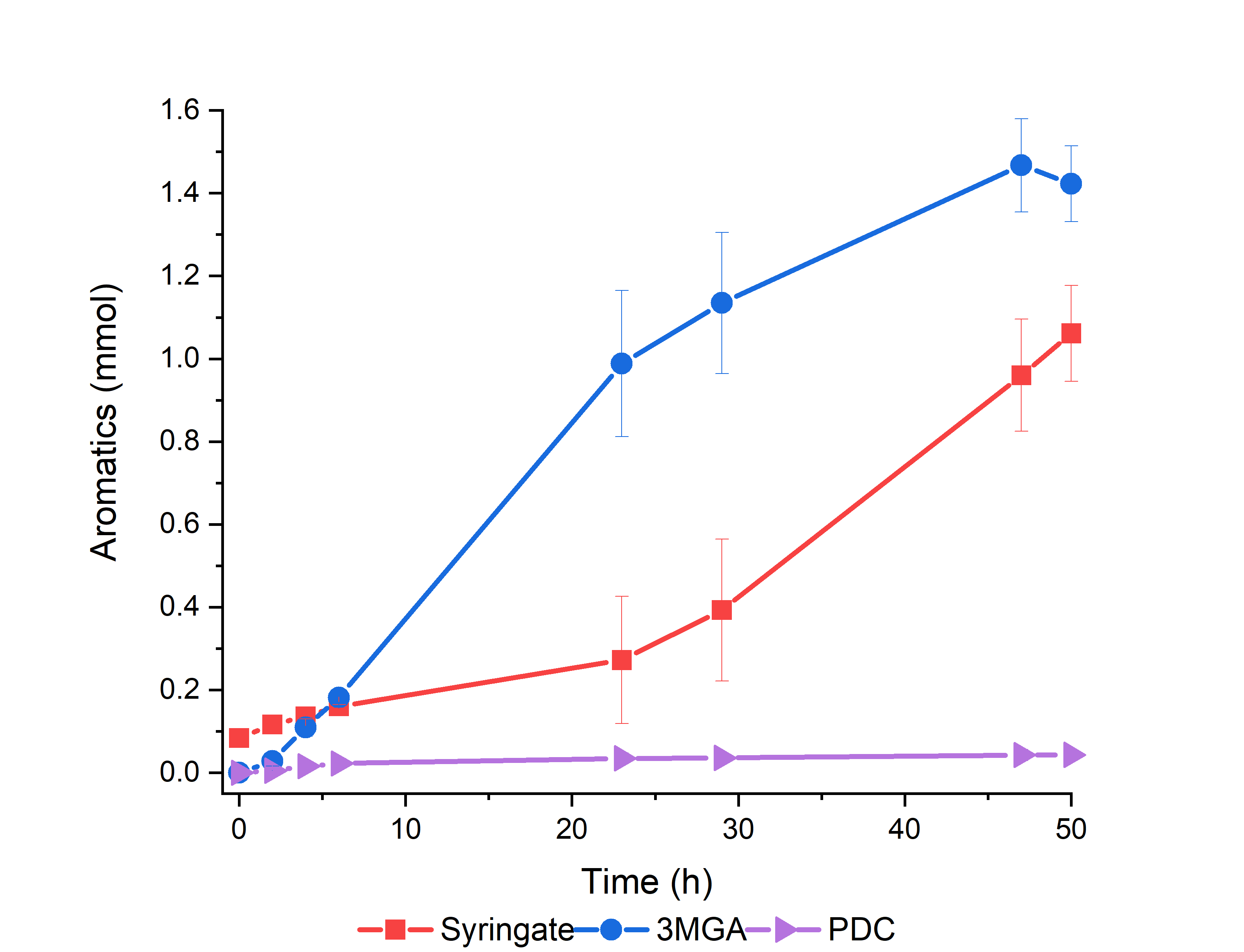


Figure S11. Production of PDC in bioreactor with strain ASA1005 with syringate and glucose feeding. The figure shows the mean values and standard deviation of two parallel cultivations. Molar quantities are based on calculated media volumes and metabolite concentrations analyzed with HPLC. Detailed culture conditions are explained in the materials and methods section.


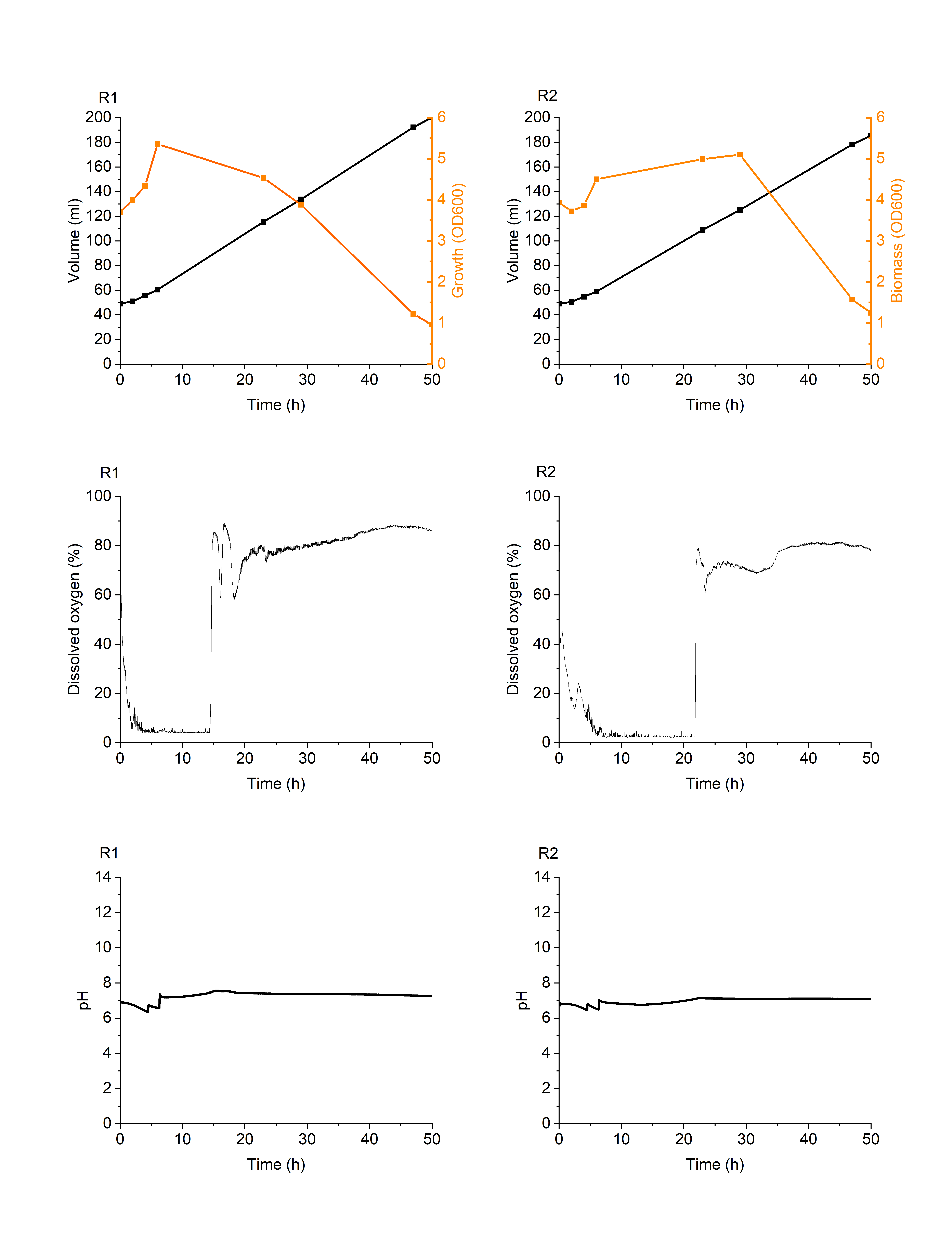


Figure S12. Bioreactor parameters from cultivation of strain ASA1005 with syringate and glucose feeding. Media volume (ml) and cell biomass (OD600) are shown in the same figure, and dissolved oxygen in a separate figure. The figures present two parallel cultivations R1 and R2. Detailed culture conditions are explained in the materials and methods section.


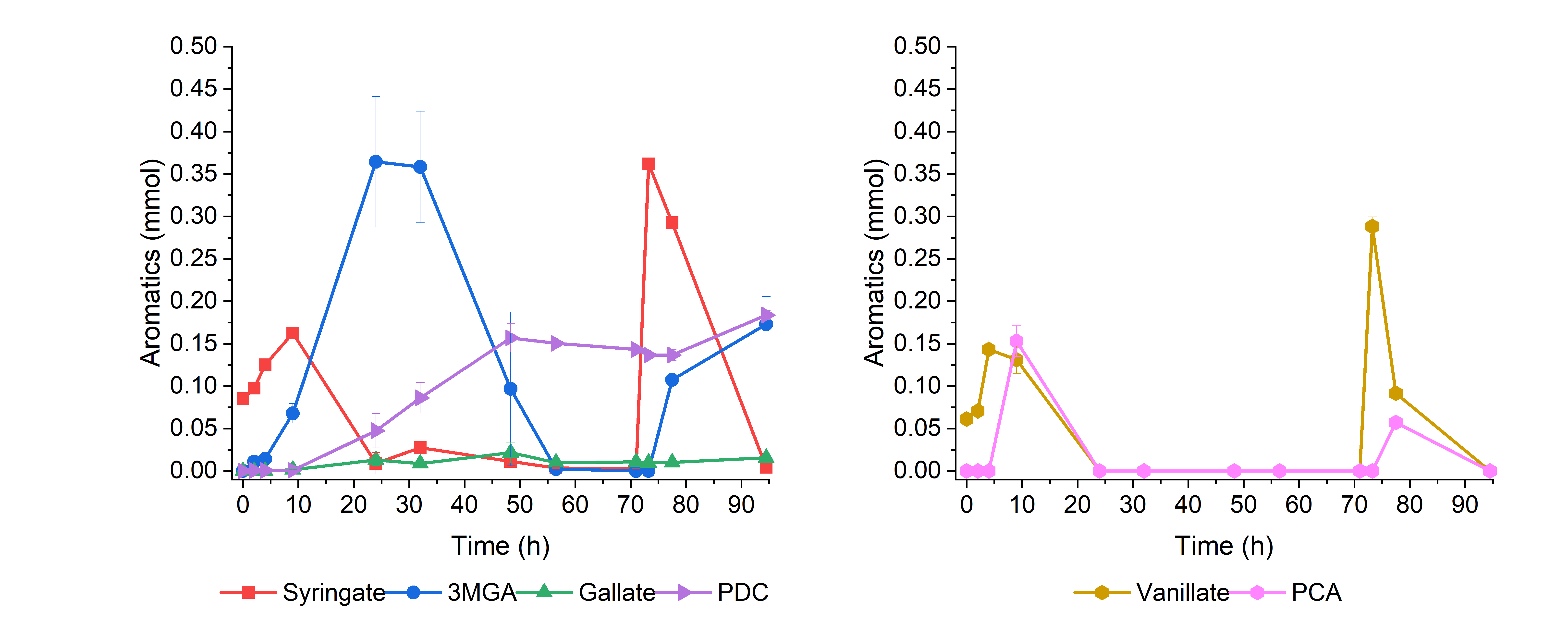


Figure S13. Production of PDC in bioreactor with strain ASA1005 with vanillate, syringate and glucose feeding. The figure show the mean values and standard deviation of two parallel cultivations. Molar quantities are based on calculated media volumes and metabolite concentrations analyzed with HPLC. Detailed culture conditions are explained in the materials and methods section. At 73 h, an additional 0.45 mmol (resulting in approximately 2 mM concentration of each) vanillate and syringate were added to the reactors. Slight increase in PDC was detected after this, but overall yield after 71 h was not improved.


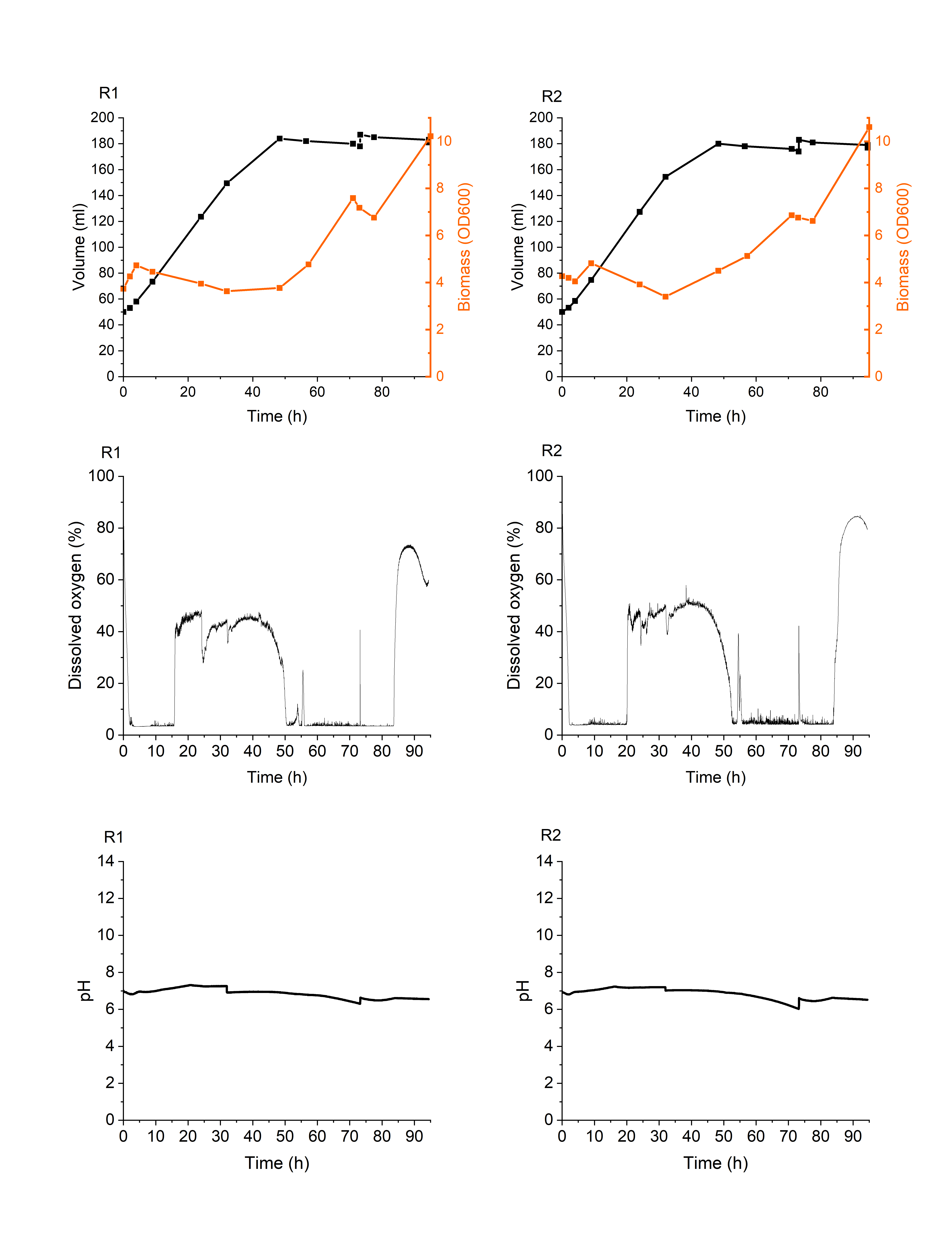


Figure S14. Bioreactor parameters from cultivation of strain ASA1005 with vanillate, syringate, and glucose feeding. Media volume (ml) and cell biomass (OD600) are shown in the same figure, and dissolved oxygen in a separate figure. The figures present two parallel cultivations R1 and R2. Detailed culture conditions are explained in the materials and methods section.

**References**

Schuster LA, Reisch CR. A plasmid toolbox for controlled gene expression across the Proteobacteria. Nucleic Acids Res. 2021 Jul 9;49(12):7189–202.
